# Targeted therapies reshape extracellular matrix remodeling and microenvironmental regulation in pediatric acute myeloid leukemia

**DOI:** 10.1007/s12672-026-04617-w

**Published:** 2026-02-21

**Authors:** Muteb Muyey Daniel, Gradel Holel Andwey

**Affiliations:** 1 Faculté de Médecine, Université de Lubumbashi (UNILU), B.P. 1825, Lubumbashi, Democratic Republic of the Congo; 2 Université Libre de Bruxelles, Franklin Roosevelt Avenue No. 50, 1050 Brussels, Belgium; 3 Present Address: International Medicine Institute, Changsha Medical University, Changsha, China

**Keywords:** Pediatric AML, TARGET-AML, TCGA-AML, GTEx, MI3454, PRMT5 inhibitor, NID1, RNA-seq, Differential expression, ECM remodeling, Gene enrichment, PPI network, Bioinformatics

## Abstract

**Background:**

Pediatric acute myeloid leukemia (AML) is a biologically distinct and aggressive malignancy with limited effective therapies. While emerging targeted agents, including menin, FLT3, and PRMT5 inhibitors, show promise, the transcriptomic effects and the role of extracellular matrix (ECM) regulators, such as nidogen-1 (NID1), in modulating therapeutic responses remain unclear.

**Methods:**

We performed integrative transcriptome analyses of pediatric AML datasets (TARGET-AML, GSE246783: MI3454; GSE292324: PRMT5 inhibition; GSE292050: NID1 knockdown) and incorporated adult AML data from TCGA and GTEx. Differential expression was evaluated using DESeq2 and limma, functional enrichment using ClusterProfiler, Metascape, and GSEA, and protein–protein interaction networks using STRING, Cytoscape, NetworkAnalyst, and GeneMANIA. Transcription factor (TF) and upstream regulator inference were conducted using DoRothEA/decoupleR, Enrichr, and TRRUST v2.

**Results:**

MI3454 treatment altered 166 genes, including upregulation of MMP10, ABCB5, and RND3, and downregulation of CD38 and ANGPT1, indicating ECM remodeling and immunomodulation. PRMT5 inhibition affected 22 RNA-processing genes, while NID1 knockdown influenced 36 ECM-related genes. GSEA consistently highlighted ECM/MMP pathway enrichment. PPI analysis identified FN1, MMP10, and EPHA7 as central hubs. TF and upstream regulator analyses revealed a shared NF-κB, CEBPB, SP1, and STAT3 axis connecting MI3454 response, PRMT5 inhibition, and NID1 disruption. Compared to prior pediatric and adult AML transcriptomic studies, this work provides novel mechanistic insights into ECM remodeling as a central mediator of therapy response.

**Conclusions:**

Targeted therapies in pediatric AML converge on ECM remodeling and a shared TF-driven regulatory axis, highlighting combinatorial strategies targeting microenvironmental and signaling vulnerabilities to overcome treatment resistance.

**Graphical abstract:**

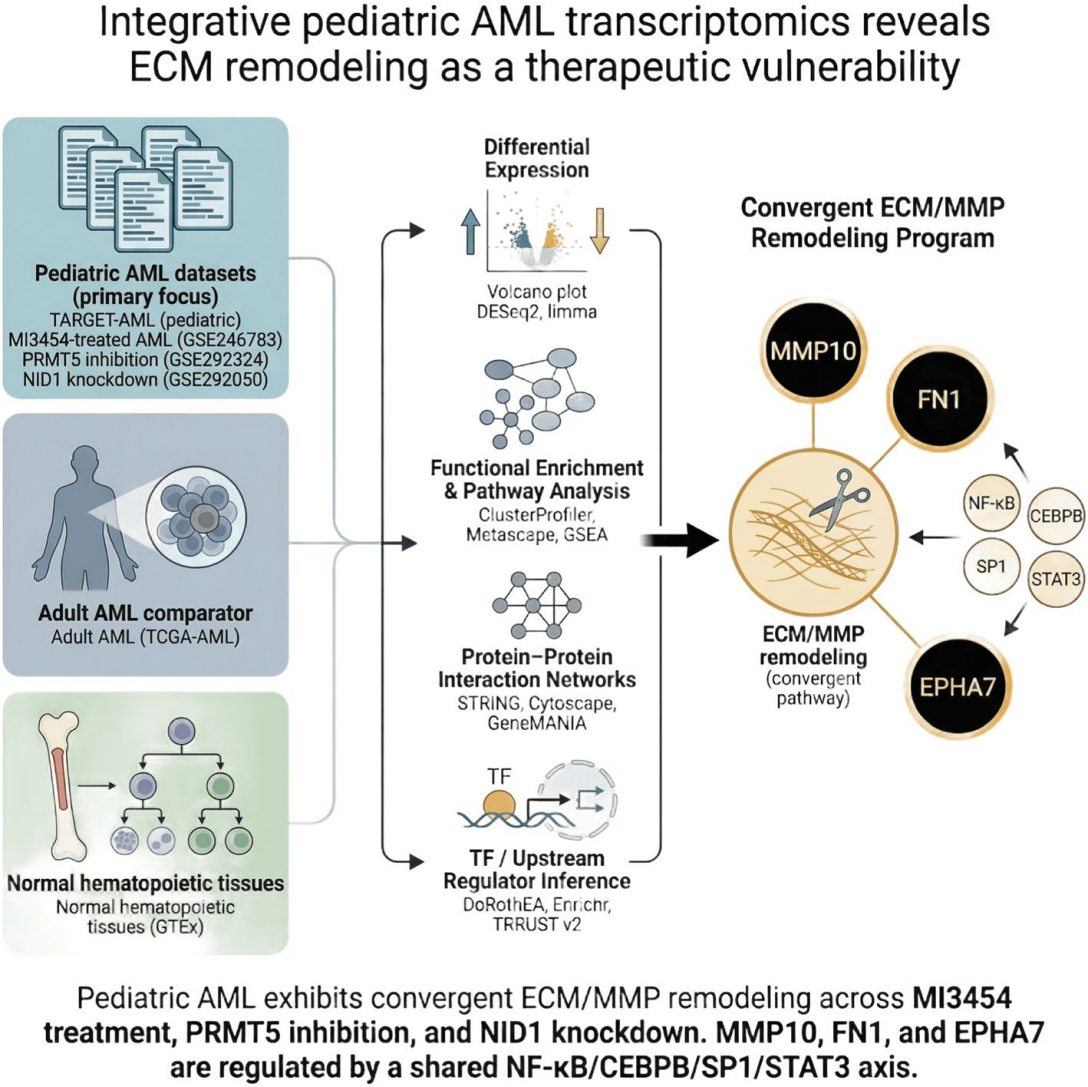

**Supplementary Information:**

The online version contains supplementary material available at 10.1007/s12672-026-04617-w.

## Introduction

Acute myeloid leukemia (AML) is a diverse hematologic malignancy characterized by the clonal proliferation of immature myeloid progenitors [1]. Pediatric AML, albeit primarily adult-onset, exhibits unique cytogenetic, mutational, and therapeutic characteristics [2–4]. Notwithstanding progress in chemotherapy and supportive care, recurrence continues to be a predominant cause of mortality, with overall survival rates falling below 70% in pediatric patients [5, 6]. The existence of therapy-resistant leukemic stem cells highlights the necessity for innovative mechanism-based treatments customized to the molecular characteristics of pediatric AML [7, 8].

Recent advancements in precision medicine have facilitated the targeting of critical oncogenic and epigenetic regulators. MI3454, a dual menin-FLT3 inhibitor, inhibits the HOXA9/MEIS1 axis and has significant anti-leukemic efficacy in FLT3-ITD AML models [9, 10]. PRMT5, an epigenetic modulator implicated in splicing, chromatin control, and the cell cycle, is often overexpressed in acute myeloid leukemia (AML) and is a viable therapeutic target [11]. Its inhibition triggers apoptosis in leukemic cells and enhances their sensitivity to other therapies [12–14]. The bone marrow microenvironment plays a crucial role in leukemogenesis and resistance to medication [12]. Nidogen-1 (NID1), a glycoprotein of the basement membrane, facilitates the formation of the extracellular matrix (ECM) and cell adhesion, and has been associated with leukemia-stroma interactions and therapeutic resistance [15]. In pediatric AML, NID1 plays a crucial role in maintaining protective marrow niches and mediating ECM-dependent survival signals, making it an attractive target for therapeutic intervention [15, 16].

Despite independent investigations of menin/FLT3, PRMT5, and NID1, their relative transcriptional effects in pediatric AML have not yet been examined. Recognizing common and distinct downstream pathways is essential for comprehending vulnerability, directing sensible medication combinations, and influencing precision treatment. While most AML transcriptomic studies have focused on adult cohorts, the contributions of ECM dynamics and the bone marrow microenvironment in pediatric AML are still poorly characterized [17–21]. Our study addresses this knowledge gap by elucidating pediatric-specific ECM and adhesion dysregulation, contrasting these findings with adult AML, and highlighting the novelty and translational relevance of our analysis.

To rectify this deficiency, we conducted an integrative transcriptome study using three publicly available RNA-seq datasets: MI3454 therapy (GSE246783), PRMT5 inhibition (GSE292324), and NID1 knockdown (GSE292050). Differential gene expression was evaluated using DESeq2, followed by pathway enrichment analysis using ClusterProfiler, Metascape, and GSEA. We utilized various analytical tools to obtain complementary insights: DESeq2 identifies statistically significant expression alterations; ClusterProfiler and Metascape conduct ontology- and pathway-level enrichment analyses; and GSEA identifies coordinated shifts in predefined gene sets, even when individual genes display modest fold-changes. Protein–protein interaction (PPI) networks and hub genes were delineated using STRING, Cytoscape, **NetworkAnalyst**, and GeneMANIA.

This work represents the **first comparative transcriptomic investigation of these three therapeutic perturbations in pediatric AML**, revealing shared dysregulation of RNA-splicing and ECM-remodeling pathways, thereby providing a mechanistic basis for potential combinatorial strategies.

## Materials and methods

### Data acquisition and preprocessing

This study leveraged three publicly available transcriptomic datasets from the Gene Expression Omnibus (GEO) to evaluate the molecular effects of targeted therapies in pediatric acute myeloid leukemia (AML):

- GSE246783: AML patient-derived cells treated with MI3454, a dual FLT3/menin inhibitor [22].
- GSE292324: Transcriptional profiling of MDSL cells exposed to PRT543, a selective PRMT5 inhibitor [23]. Included solely to assess the conservation of PRMT5-responsive transcriptional programs across myeloid malignancies.
- GSE292050: Assessment of gene expression changes following NID1 knockdown in leukemia models, emphasizing extracellular matrix (ECM) remodeling [24].

Raw count matrices for GSE246783 and GSE292050 were processed in **R version 4.2.3,** utilizing the **DESeq2 package version 1.38.3**. The GSE292324 dataset, presented as normalized expression values, was analyzed using limma (v3.54.1) with empirical Bayes moderation. GSE292324 was analyzed independently and not pooled with pediatric datasets, thereby avoiding potential cross-cohort artifacts. Gene annotation adhered to the **GENCODE v41** reference. Genes exhibiting low expression (fewer than 10 readings in at least 80% of the samples) were excluded. Ensembl identifiers were correlated with HGNC gene symbols using the org.Hs.eg.db package to enhance interpretability and enable cross-dataset comparisons.

Pediatric AML and adult MDSL share fundamental AML processes, such as RNA splicing dysregulation, stemness, and inflammatory signaling [25, 26]. Conservation of transcriptional effects was assessed through sign-coherence of gene-level log₂ fold-change values and parallel enrichment of ECM remodeling, adhesion, and splicing pathways (Supplementary Tables S1–S3; Figure S1). Principal component analysis (PCA) confirmed sample-level variation and evaluated dataset-specific batch effects. This approach ensures that mechanistic insights from PRMT5 inhibition in adult MDSL cells remain informative for understanding conserved transcriptional programs relevant to pediatric AML.

A comprehensive overview of all datasets used in this study, including GEO accession numbers, study context, sample counts, experimental conditions, treatment–control comparisons, and pediatric status, is provided in Supplementary Table Sx. This summary ensures full dataset traceability and supports compliance with FAIR (Findable, Accessible, Interoperable, and Reusable) data principles.

### Differential gene expression analysis

Differential expression analysis was performed independently for each dataset. **DESeq2** was utilized for raw count data (GSE246783, GSE292050), whereas **limma** was employed for pre-normalized data (GSE292324). Genes exhibiting a log₂ fold change of at least 1 and a Benjamini–Hochberg corrected *p*-value of less than 0.05 were categorized as substantially differentially expressed (DEGs). To ensure robustness, sensitivity analyses were performed using a more permissive |log₂FC|≥ 0.58 (1.5-fold change), confirming the consistency of pathway-level conclusions.

For cross-dataset comparisons, DEGs were assessed using **log**_**2**_**FC sign-coherence and normalized enrichment scores (NES) from GSEA**, allowing identification of conserved pathways across datasets without introducing batch artifacts.

To visually summarize differential expression and sensitivity, a composite supplementary figure (Supplementary Figure S2) was generated. For datasets with available log₂FC (PRMT5i), standard volcano plots were plotted; for datasets lacking log₂FC (MI3454, NID1 KD), ranked –log10(FDR) panels were used. The bottom panel summarizes median DEG counts under replicate down-sampling to assess power and robustness across perturbations. This figure illustrates relative DEG yield, harmonized thresholds, and the persistence of ECM/adhesion enrichment across resampling, supporting that observed differences are driven by biological scope rather than analytical thresholds or sample size.

All datasets were analyzed separately rather than combined, and cross-dataset comparisons focused only on consistent log₂FC direction and pathway enrichment to minimize batch effects. The DEG lists were created for subsequent enrichment and network-based analyses [27–29].

### Visualization of differential expression patterns

Volcano plots were created using **EnhancedVolcano (v1.16.0**) to illustrate the importance and effect magnitude of differentially expressed genes (DEGs) [30]. Principal component analysis (PCA) was employed to investigate sample-level variance and possible batch effects. Hierarchical clustering and heat maps were generated using **Pheatmap (v1.0.12)** to illustrate the expression profiles of the most significant DEGs across various circumstances [31].

PCA was specifically used to evaluate potential batch effects between datasets, confirming that independent analyses captured dataset-specific variance while enabling cross-dataset comparisons at the pathway level.

### Gene ontology and KEGG pathway enrichment analysis

Functional annotation of differentially expressed genes (DEGs) was conducted utilizing **clusterProfiler (v4.6.0).** Over-representation analysis (ORA) identified enriched **Gene Ontology (GO)** terms, Biological Process (BP), Molecular Function (MF), and Cellular Component (CC), along with **KEGG** pathways. Significance was established as an FDR-adjusted p < 0.05. Results are represented by dot plots, bar charts, and enrichment networks [32].

Cross-dataset integration was performed at the **pathway level**, comparing NES correlation and log₂FC directionality to identify biologically conserved AML programs, such as ECM remodeling and RNA splicing.

### Gene set enrichment analysis (GSEA)

GSEA was conducted on pre-ranked log₂ fold-change values using **fgsea (v1.24.0)** to assess coordinated alterations at the pathway level [33]. Cross-dataset GSEA integration was performed by comparing log₂FC directionality (sign-coherence) and correlating normalized enrichment scores (NES) across datasets, rather than pooling expression values. Normalized enrichment scores (NES) and FDR q-values were reported as continuous metrics following standard conventions (FDR q < 0.25 for discovery, q < 0.05 emphasized where appropriate), avoiding arbitrary ES categorization. The selected gene sets of interest encompassed ECM/MMP remodeling, epithelial–mesenchymal transition (EMT), and RNA processing pathways. Conserved pathways were highlighted when NES enrichment and log₂FC directionality were consistent across datasets, emphasizing biologically conserved transcriptional programs. Enrichment plots depict the cumulative distribution of genes in each collection [34, 35]. Conserved pathways were defined by consistent NES and log₂FC directionality across datasets, emphasizing biologically shared transcriptional programs without merging raw expression matrices.

### Transcription factor and upstream regulator analysis

To delineate the regulatory architecture underlying transcriptional responses to MI3454 treatment, PRMT5 inhibition, and NID1 knockdown, we performed an integrated transcription factor (TF) and upstream regulator analysis combining regulon activity inference, motif/ChIP-based enrichment, and curated TF–target knowledge bases. This multilayered strategy was designed to identify both direct regulatory drivers and higher-order regulatory programs shared across perturbations.

1. *TF–Target Enrichment (Enrichr and RcisTarget)*
  Differentially expressed genes (DEGs) from each dataset were interrogated using Enrichr (ChEA, ENCODE, and ChIP-X libraries) to quantify the overrepresentation of TF binding signatures. Enrichment significance was assessed using FDR-adjusted *p*-values, combined scores, and observed–expected target overlaps.
  In parallel, promoter- and motif-level enrichments were evaluated using RcisTarget (hg19 motif collection). Normalized enrichment scores (NES) > 3.0 were considered significant motif-regulatory signals.
2. *TF Activity Inference (DoRothEA/decoupleR)*
  To estimate TF activity beyond simple target enrichment, we computed regulon activity scores using DoRothEA (confidence levels A–C) implemented in the decoupleR pipeline.
  For each TF, normalized enrichment scores (NES) were generated using a weighted-mean model incorporating signed TF–target interactions. Null distributions were derived through permutation or empirical resampling, and TFs with adjusted *p*-values < 0.05 were classified as significantly activated or repressed. This allowed us to distinguish true regulatory activation from passive target co-expression.
3. *Upstream Regulator Validation (TRRUST v2)*
  To anchor predictions in experimentally supported biology, all candidate TFs were cross-referenced with TRRUST v2, which provides curated TF–target interactions and activation/repression directionality data. The over-representation of TRRUST targets among DEGs was tested using Fisher’s exact test with FDR correction.
4. *Integration and Robust TF Definition*
  TFs were considered **robust upstream regulators** when they satisfied both criteria:
  **FDR < 0.05**, and
  **Independent support from ≥ 2 analytical frameworks** (DoRothEA, RcisTarget/Enrichr, TRRUST) or ≥ 2 datasets (MI3454, PRMT5 inhibition, and NID1 knockdown).
  To synthesise the results across datasets, we constructed a TF–target regulatory network in which:
  **Nodes** represent TFs and DEGs.
  **Edges** represent validated TRRUST interactions or high-confidence motif- or ChIP-supported predictions.
  **Node attributes** encode functional module assignment (ECM/MMP vs. immune/inflammatory) and dataset specificity (MI3454-specific, PRMT5-specific, NID1-specific, or shared).
  Network visualization was performed using a force-directed layout, and TF robustness was summarized in a schematic overlap diagram (Fig. 8B–D).
5. *Context-Specific Prioritization: PRMT5 and NID1*
  Because PRMT5 functions primarily in chromatin organization and RNA processing, we specifically evaluated enrichment of splicing-associated TFs and RNA-binding factors (e.g., SRSF family, HNRNP family, MYC).

For NID1 knockdown, emphasis was placed on ECM- and adhesion-associated regulators (e.g. ETS1, TEAD1/TEAD4, and SMAD family) and their integration with the MI3454-responsive ECM network.

#### Summary of outputs

This integrated strategy consistently identified a core set of convergent regulators, including NF-κB (NFKB1/RELA), STAT3, SP1, and CEBPB, which were enriched, transcriptionally active, and validated by curated TF–target interactions across ≥ 2 perturbations. These TFs formed the backbone of the ECM–immune regulatory circuit explored in the Results and Discussion sections.

### Protein–protein interaction (PPI) network construction

To clarify putative protein interaction landscapes, PPI networks were constructed using the **STRING database (v11.5)** with a minimum interaction confidence score of 0.4 [36]. Networks were depicted in **Cytoscape (version 3.9.1)** [37]. **CytoHubba (v1.6.1)** was utilized to locate central hub genes by Maximal Clique Centrality (MCC) [38, 39]. Densely interconnected network modules were identified using MCODE, facilitating the discovery of functionally cohesive clusters.

### Functional gene–gene network analysis via GeneMANIA and metascape

**GeneMANIA** was employed to forecast functional gene–gene relationships based on co-expression, co-localization [40], and common pathways. Complementary analyses were performed using **Metascape (v3.5.20250101)** to investigate GO, KEGG, and Reactome pathway enrichment, annotate molecular clusters, and create similarity-based networks, offering a comprehensive perspective on the regulatory landscape of each therapy [41].

### Pathway mapping and visualization

Significant DEGs were aligned with canonical **KEGG** pathways utilizing the **Pathview program (v1.38.0)** [42]. Pathway reconstructions have focused on **FLT3 signaling**, **HOXA cluster control**, and **ECM remodeling** to elucidate molecular changes within physiologically pertinent circuits.

### Ethical considerations

All analyses were performed using publicly accessible, de-identified RNA-seq datasets available from the NCBI GEO repository. This study did not require ethical approval or informed permission because it did not involve studies on human or animal subjects. The utilization of data adhered to the stipulations and license agreements of the original data providers.

### Software and computational tools

**Table:** 

Tool/Package	Version	Application
R	4.2.3	Statistical computing
DESeq2	1.38.3	Differential expression (count data)
limma	3.54.1	Differential expression (normalized data)
clusterProfiler	4.6.0	GO/KEGG enrichment analysis
fgsea	1.24.0	Gene set enrichment analysis
EnhancedVolcano	1.16.0	Volcano plot generation
pheatmap	1.0.12	Heatmap visualization
Cytoscape	3.9.1	Network construction and visualization
STRING	11.5	PPI interaction data
CytoHubba	1.6.1	Hub gene identification
GeneMANIA	–	Gene–gene interaction prediction
Metascape	v3.5.20250101	Functional enrichment and network clustering
Pathview	1.38.0	Pathway expression mapping

### In silico validation of hub genes in independent AML cohorts

#### Data sources

Independent validation was performed using publicly available datasets from the **TCGA-AML** cohort (adult acute myeloid leukemia) and normal bone marrow samples from the **GTEx** database, accessed through the **GEPIA** web interface (http://gepia.cancer-pku.cn).

Pediatric AML survival trends were conceptually cross-checked using **TARGET-AML** summaries; however, all statistical analyses and figures presented here reflect **TCGA-AML versus GTEx** comparisons unless otherwise specified [43].

#### Gene selection

The hub genes **FN1**, **MMP10**, and **EPHA7** were selected based on their network centrality, consistent dysregulation across discovery analyses, and known biological roles in **extracellular matrix (ECM) remodeling** and **ephrin signaling**.

#### Expression analysis (AML vs. normal)

Expression differences between AML and normal samples were evaluated using log₂-transformed **TPM** values (GEPIA standard normalization). Data were visualized using combined **violin and box plots** to display the median, interquartile range, and kernel density.

Statistical significance was determined using **GEPIA’s two-sided tests**, with *p*-values reported as *p* < 0.01 where applicable.

#### Survival analysis (overall survival)

Overall survival (OS) was analyzed using **Kaplan–Meier** estimates with **log-rank testing** under default GEPIA parameters. Patients were dichotomized into “High” and “Low” expression groups based on median cutoffs.

Kaplan–Meier curves were plotted with **95% confidence intervals** and accompanying **risk tables**. Consistent with the portal outputs, higher **FN1** expression was associated with **poorer OS**, whereas lower **EPHA7** expression predicted **better OS**.

#### Figure generation

GEPIA-style figures were generated for visual consistency, including:

- Expression plots (AML vs. Normal) for **FN1** and **MMP10**
- Kaplan–Meier survival plots for **FN1** and **EPHA7**

All panels were formatted with uniform color schemes, legends, and statistical annotations. High-resolution **PNG** files were prepared for inclusion in the main manuscript and supplementary materials.

#### Reproducibility and limitations

No raw TCGA or GTEx data were downloaded locally; all results were derived from **GEPIA-processed outputs**. Minor variations in sample sizes or effect estimates may occur due to periodic portal updates. All analyses are fully reproducible by re-querying GEPIA or TARGET using the provided gene identifiers.

## Results

### Differential gene expression analysis reveals distinct molecular responses

**Fig. 1:**
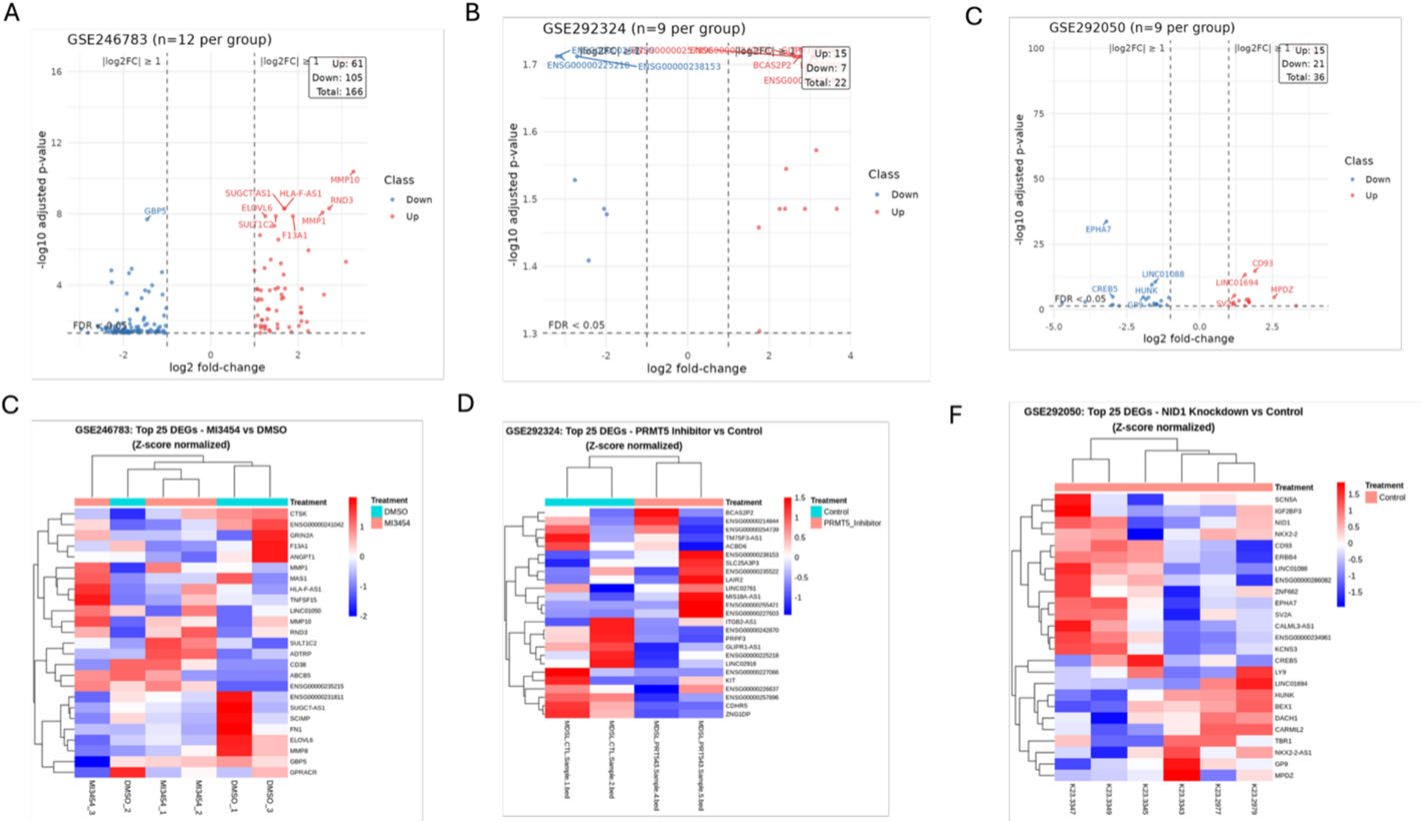
Transcriptomic profiling of pediatric AML datasets. Volcano plots (**A**–**C**) show differential expression for GSE246783 (MI3454 vs. DMSO), GSE292324 (PRMT5 inhibitor vs. control), and GSE292050 (NID1 knockdown vs. control). The x-axis represents log₂ fold-change and the y-axis represents − log₁₀ adjusted *p*-value (FDR). Vertical dashed lines indicate |log₂FC|= 1 and the horizontal dashed line indicates FDR = 0.05. Points are colored by significance class: Up (red), Down (blue), NS (gray). Insets report counts of Up, Down, and total significant DEGs, and the top 10 most significant genes are labeled. Sample sizes per group: n = 12 (GSE246783) and n = 9 (GSE292324, GSE292050). Heatmaps (**D**–**F**) display the top 25 DEGs for each dataset, with rows representing genes and columns representing biological replicates. Expression values are row-scaled (Z-score) to highlight relative changes. Hierarchical clustering was performed using Euclidean distance and complete linkage. Color scale: blue = low expression, red = high expression. Sample sizes per group correspond to those in the volcano plots

To evaluate the impact of targeted therapies on the transcriptome of pediatric acute myeloid leukemia (AML), we examined three distinct RNA-seq datasets corresponding to various perturbations: FLT3/menin dual inhibition (MI3454; GSE246783), PRMT5 inhibition (PRT543; GSE292324), and NID1 knockdown (GSE292050). MI3454 elicited the most significant changes in the transcriptome (Fig. 1A–C).

MI3454 therapy resulted in 166 differentially expressed genes (DEGs) (adjusted p < 0.05, |log₂FC|≥ 1), including 61 upregulated and 105 downregulated genes (Supplementary Table S1). Upregulated genes, including ABCB5, MMP10, and RND3, are associated with multidrug resistance, matrix remodeling, and cytoskeletal dynamics. In contrast, the downregulated genes CD38, ANGPT1, and GBP5 indicated a reduction in immunological activation and angiogenic pathways.

Supplementary Figure S2 provides a comparative overview of differential expression across perturbations, showing volcano plots or ranked –log₁₀(FDR) distributions (MI3454, PRMT5i, NID1 KD) and median DEG counts under down-sampling, confirming that observed differences arise primarily from biological scope rather than analytical thresholds or replicate imbalance.

Inhibition of PRMT5 by PRT543 resulted in minor transcriptional alterations, yielding only 22 differentially expressed genes (DEGs) (Supplementary Table S2), predominantly long non-coding RNAs (lncRNAs), such as TM7SF3-AS1 and GLIPR1-AS1, suggesting specific epigenetic influences (Fig. 1B). These findings highlight PRMT5's function in modulating non-coding RNA expression and chromatin structure.

Knockdown of NID1 modified 36 genes (Supplementary Table S3), significantly impacting ephrin signaling and cellular motility. Genes such as EPHA7 and HUNK exhibit considerable dysregulation, indicating disturbances in intracellular trafficking and cytoskeletal control. Moreover, overexpression of NID1, CD93, and LINC01694 indicated compensatory mechanisms associated with extracellular matrix integrity and adhesion (Fig. 1C).

#### Transcriptomic signatures correlate with treatment-specific pathways

Heatmaps of the top 25 differentially expressed genes per condition further elucidated the distinct transcriptional profiles elicited by each intervention (Fig. 1D–F). MI3454 prompted extensive transcriptional reprogramming, aligning with simultaneous inhibition of FLT3 and menin. Suppression of PRMT5 mostly affected lncRNAs, strengthening their epigenetic regulatory function. The suppression of NID1 predominantly influences extracellular matrix remodeling, cellular adhesion, and ephrin receptor signaling, which are key characteristics of stromal interaction and leukemic cell plasticity refer to Table 1.

**Table 1:** Transcriptomic impact of different interventions in AML cells

Dataset	DEGs (abs log2FC ≥ 1, padj < 0.05)	Percent of Genes	Interpretation
MI3454 (GSE246783)	166 genes	0.84%	Broad signaling and transcriptional inhibition
PRMT5 inhibitor (GSE292324)	22 genes	0.15%	Precise epigenetic modulation via lncRNAs
NID1 Knockdown (GSE292050)	36 genes	0.12%	ECM remodeling and cell adhesion dynamics

DEGs, differentially expressed genes; Percent of Genes, proportion of all expressed genes in the dataset that meet |log2FC| ≥ 1 and padj < 0.05, reflecting the extent of transcriptional response for each treatment

Collectively, these data highlight the transcriptional variability caused by different molecular disruptions in AML cells, each indicative of the mechanism of action of the targeted pathway. The substantial transcriptional reprogramming associated with MI3454 underlines its extensive inhibitory effect on leukemogenic signaling, whereas the targeted response to PRMT5 and NID1 disruption highlights specialized regulatory networks that control AML pathobiology.

### Transcriptional landscape and functional enrichment of ECM/MMP signatures across targeted interventions

To clarify the transcriptional reconfiguration of extracellular matrix (ECM) remodeling and matrix metalloproteinase (MMP) signaling under specific perturbations, we conducted Gene Ontology (GO) and pathway enrichment analyses utilizing pre-ranked gene lists obtained from three distinct RNA-seq datasets: GSE246783 (MI3454-treated pediatric AML), GSE292324 (PRMT5 inhibition), and GSE292050 (NID1 knockdown). A selected panel of canonical ECM/MMP genes, comprising MMP1, MMP3, MMP9, MMP10, MMP13, COL1A1, COL1A2, PLAU, and PLAUR, was employed to facilitate pathway analysis.

**Fig. 2:**
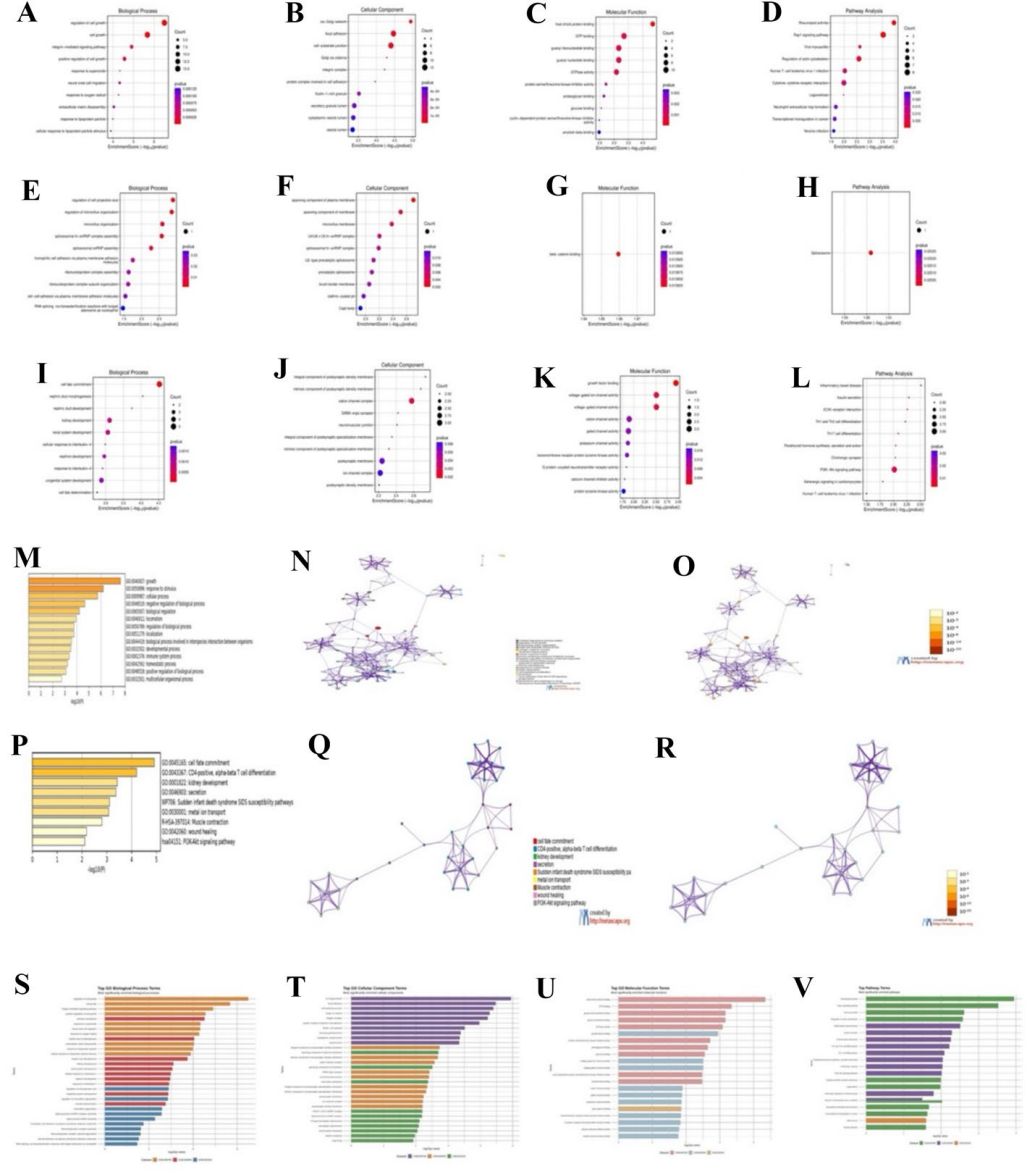
Quantitative GO and pathway enrichment analyses across AML perturbations. **A**–**D** MI3454 (GSE246783): Top 10 enriched GO terms (BP, CC, MF) and KEGG pathways. Bubble/bar size = gene count; color gradient = –log10(adj. *p*-value). Numerical labels indicate gene count, fold enrichment, ES. Key enrichments: ECM organization (adj. p = 1.2e–5, ES = 2.45, count = 12), cytokine activity (adj. p = 3.1e–4, ES = 1.92, count = 9). **E**–**H** PRMT5 inhibition (GSE292324): Top GO/KEGG terms; notable enrichments: RNA splicing (adj. p = 2.8e–5, ES = 2.31, count = 15). **I**–**L** NID1 KD (GSE292050): Top GO/KEGG terms; ECM pathways enriched: ECM-receptor interaction (adj. p = 5.5e–3, ES = 2.26, count = 2). **M**, **P** Heatmaps of GO term enrichment via Metascape; numeric annotation = gene count. **N**, **Q** Functional enrichment networks; node size = gene count, node color = cluster identity. Key clusters: ECM remodeling, collagen fibril assembly, RNA splicing. **O**, **R** Term significance networks; node size = gene content; color = –log10(adj. *p*-value). **S**–**U** Comparative enrichment plots: top 30 GO terms across datasets. **V** KEGG summary: 21 shared or condition-specific pathways; bubble size = gene count, color gradient = –log10(adj. *p*-value). Selected pathways include ECM-receptor interaction, PI3K-Akt signaling, cytokine–cytokine receptor interaction

Differential gene expression analysis conducted with DESeq2 (adjusted p < 0.05, |log₂FC|> 1) indicated significant transcriptional alterations in ECM/MMP components across datasets (Fig. 4K), implying pathway-specific responses to each molecular intervention. Gene Ontology (GO) and pathway enrichment analyses were performed to elucidate the biological significance of differentially expressed genes (DEGs), with findings presented in Fig. 2 and Supplementary Tables S4–S6.

In the MI3454-treated AML model (GSE246783), enriched GO keywords were associated with ECM organization, cytokine activity, and immunological modulation (Fig. 2A–D), signifying a significant modification of the leukemic milieu. The inhibition of PRMT5 (GSE292324) revealed a unique enrichment profile, mostly associated with RNA splicing machinery and the spliceosome (Fig. 2E–H), aligning with PRMT5's function in transcriptome control. Conversely, NID1 knockdown (GSE292050) resulted in significant enrichment of ECM-receptor interaction and collagen fibril assembly pathways (Fig. 2I–L), highlighting NID1’s functional involvement in preserving ECM integrity.

Supplementary analysis using Metascape further corroborated these findings. Heatmaps (Fig. 2M, P), network visualizations (Fig. 2N, Q), and term significance plots (Fig. 2O, R) depicted unique and overlapping biological themes across datasets, demonstrating strong convergence in ECM-related signaling and divergence in RNA processing and matrix assembly characteristics.

#### Functional enrichment summary

- *GO Biological Process (BP):* Thirty significantly enriched biological processes were found (Fig. 2S), encompassing the regulation of cell proliferation, integrin-mediated signaling, and extracellular structural organization, with *p*-values ranging from < 1e-7 to < 0.05.
- *GO Cellular Component (CC):* Analysis of 30 subcellular localizations (Fig. 2T) demonstrated significant connections with extracellular matrix structures, plasma membrane regions, and vesicular transport.
- *GO Molecular* Function (MF): Twenty-one functional categories were enriched, including proteolytic activity, cytokine receptor binding, and cell adhesion (Fig. 2U).
- *KEGG Pathways:* Twenty-one signaling pathways were considerably enriched (Fig. 2V), including ECM-receptor contact, PI3K-Akt signaling, and cytokine-cytokine receptor interaction, thereby underscoring critical oncogenic and microenvironmental circuits.

#### Integrated interpretation

This comparative enrichment analysis across datasets revealed both conserved and context-specific transcriptomic mechanisms. ECM/MMP remodeling and immunological signaling emerged as significant disruption pathways, while PRMT5 inhibition particularly stimulated the spliceosomal machinery, and NID1 knockdown hindered collagen organization. These findings improve the comprehension of the therapeutic modulation of the leukemic milieu and underscore novel targets for selective pharmacological combinations in AML.

### GSEA reveals ECM/MMP pathway activation as a shared molecular signature of MI3454 treatment and NID1 suppression

**Fig. 3:**
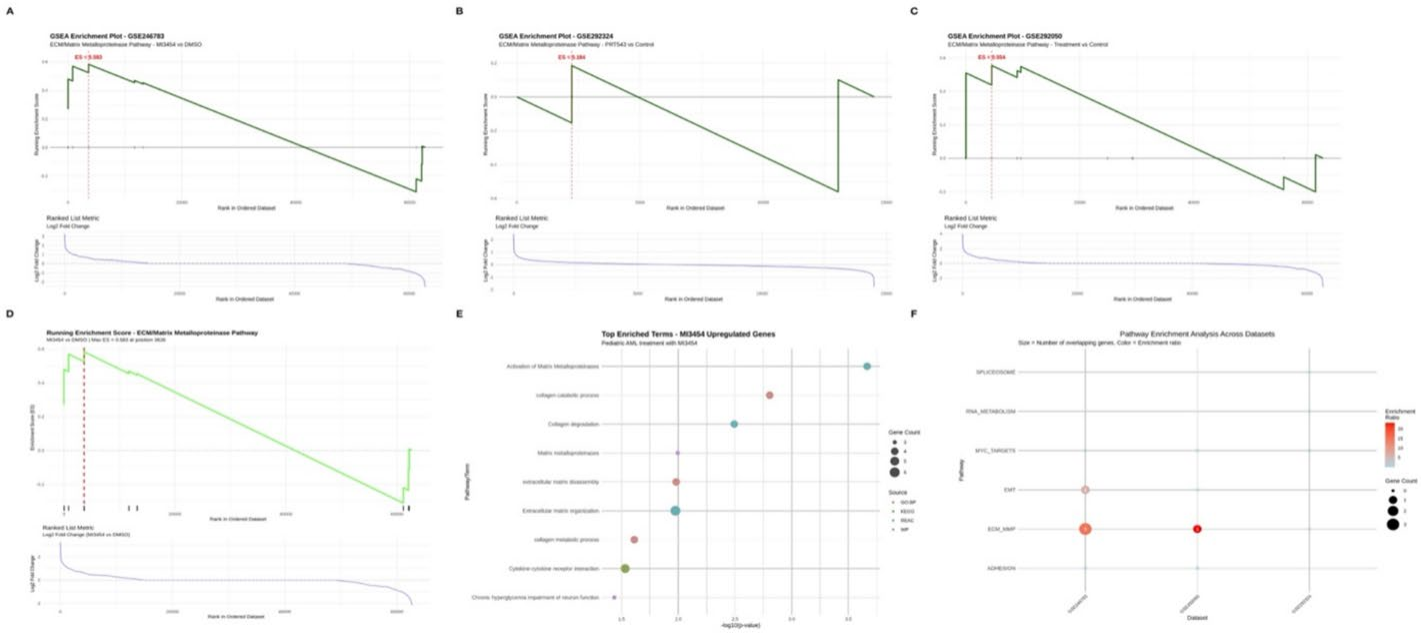
GSEA reveals ECM/MMP pathway activation across AML perturbations. **A**–**C** Enrichment plots of the ECM/MMP gene set in **MI3454-treated cells (GSE246783)**, **PRMT5 inhibition (GSE292324)**, and **NID1 knockdown (GSE292050)**. Normalized counts were used for ranking; enrichment scores (ES) and fgsea-calculated *p*-values are indicated (MI3454: ES = 0.5828, 100% coverage; NID1 KD: ES = 0.5538, 100% coverage; PRMT5i: ES = 0.1845, 22% coverage). D Ranked enrichment curve for ECM/MMP genes in MI3454-treated cells, with key contributors labeled (MMP10, MMP1, COL1A2); positive ES values indicate pathway activation. **E**–**F** Comparative summary of ECM/MMP pathway enrichment across MI3454 and NID1 KD datasets, highlighting upregulation of FN1, MMP10, and other ECM components. (*p < 0.05, **p < 0.01, ***p < 0.001)

To investigate pathway-level alterations, Gene Set Enrichment Analysis (GSEA) was conducted using a curated gene set consisting of canonical ECM/MMP components. Both MI3454 therapy and NID1 knockdown exhibited significant positive enrichment for the ECM/MMP pathway; however, PRMT5 inhibition displayed minimal enrichment (Fig. 3A–C).

In MI3454-treated cells, all nine genes in the ECM/MMP set were highly elevated, with **MMP10, MMP1**, and **COL1A2** predominantly contributing to the enrichment signal (ES = 0.5828, 100% coverage). Knockdown of NID1 resulted in a comparable enrichment profile (ES = 0.5538, 100% coverage), indicative of compensatory extracellular matrix remodeling. Conversely, suppression of PRMT5 resulted in minimal enrichment (ES = 0.1845), with only **PLAU** and **PLAUR** exhibiting minor upregulation (Fig. 3D) refer to Table 2.

**Table 2:** ECM/MMP pathway enrichment across AML perturbations

Dataset	Enrichment Score (ES)	Genes Found/Total	Interpretation
GSE246783 (MI3454)	**0.5828**	9/9 (100%)	Strong ECM/MMP activation
GSE292050 (NID1 KD)	**0.5538**	9/9 (100%)	Strong ECM/MMP activation
GSE292324 (PRMT5i)	0.1845	2/9 (22%)	Weak engagement of ECM/MMP genes

ES, enrichment score calculated by GSEA; Genes Found/Total, number of ECM/MMP gene set members detected in the dataset over the total gene set size, expressed as percentage coverage; interpretation reflects the degree of ECM/MMP pathway activation for each perturbation

The enrichment map for MI3454 treatment exhibited pronounced clustering of pathway genes at the upregulated end of the ranked gene list (Fig. 3D), with **MMP10** (rank 1, log₂FC = 3.24) and **MMP1** (rank 10, log₂FC = 2.50) as the principal contributors.

#### Enrichment summary confirms ECM remodeling as a therapeutic axis

Extracellular matrix (ECM) remodeling was consistently identified as a dominant biological process by enrichment analyses across datasets. Notably, ECM-structural and matrix metalloproteinase (MMP) genes, such as FN1 and MMP10, were upregulated, especially in MI3454 and NID1 knockdown perturbations (Fig. 3E–F). These results suggest a common impairment of adhesion signaling and ECM organization downstream of NID1 silencing and FLT3/menin inhibition.

While direct functional assays, such as those involving collagen remodeling or cell adhesion and invasion, were not performed in this study, we enhanced our analysis through in silico validation using independent AML cohorts, specifically TARGET-AML and TCGA.FN1 and MMP10 exhibited persistent overexpression and strong correlations with adverse survival outcomes in these populations, underscoring their biological and clinical significance (refer to Sect. 3.7; Fig. 7).

Cross-cohort studies and transcriptome data integration highlight the importance of ECM/MMP modification as a potential therapeutic target in pediatric AML. To clarify its molecular function in leukemogenesis, this finding requires additional experimental validation using matrix remodeling and cell adhesion experiments.

### PRMT5 perturbation reveals divergent pathway signatures with convergent immune and ECM remodeling effects

To investigate the molecular effects of PRMT5 disruption, we performed pathway enrichment analyses using three RNA-seq datasets representing both pharmacological and genetic suppression: MI3454 treatment (GSE246783), NID1 knockdown (GSE292050), and PRMT5 knockdown (GSE292324).

#### Differential expression and pathway activation

**Fig. 4:**
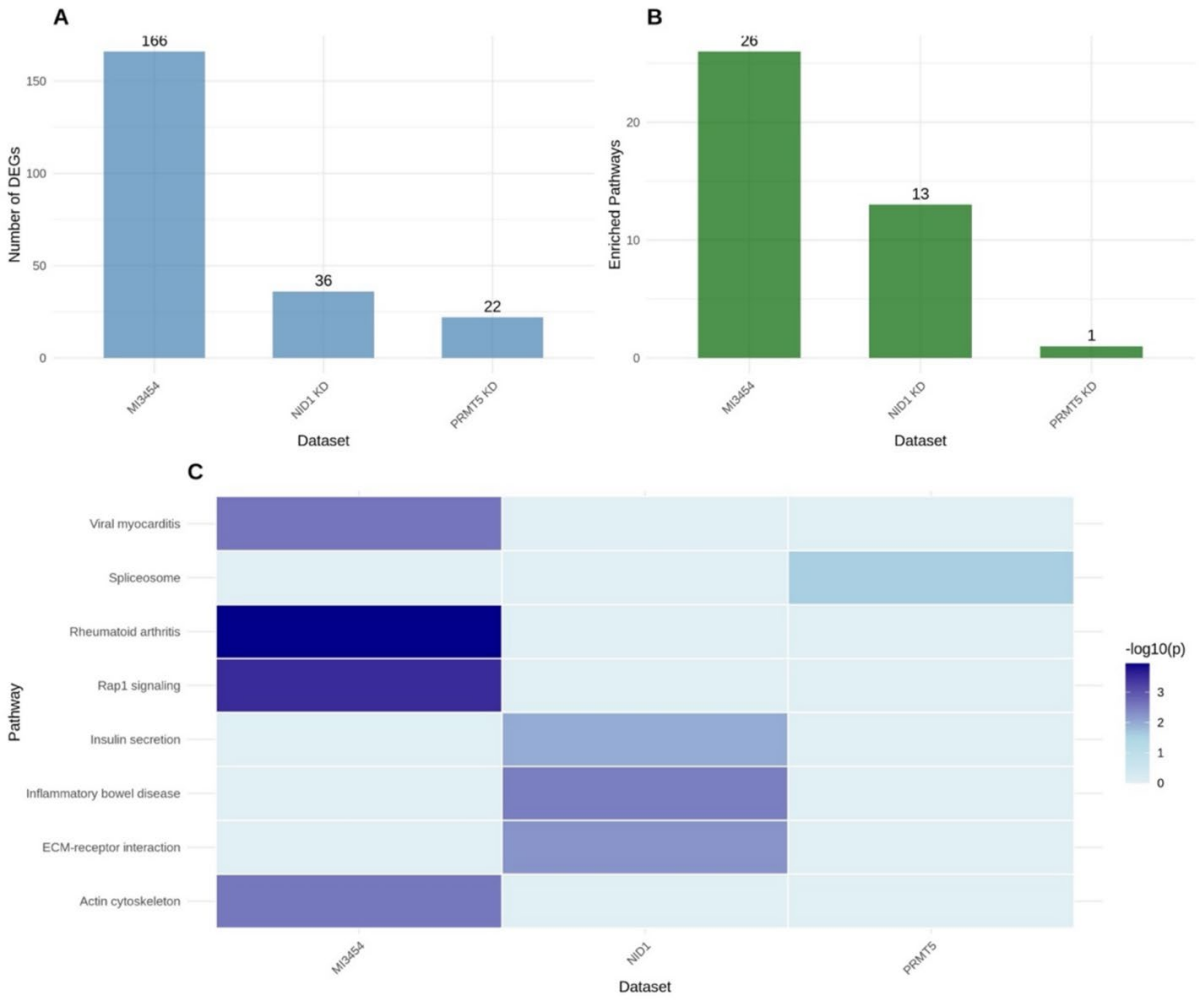
Comparative differential expression and pathway enrichment.** A** Number of DEGs per dataset (|log₂FC|≥ 1, adj. p < 0.05); **B** Number of significantly enriched KEGG pathways (p < 0.05);** C** Heatmap of pathway enrichment significance (–log10 *p*-value), where white indicates non-significant and darker blue indicates higher significance, with key pathways annotated. (*p < 0.05, **p < 0.01, ***p < 0.001)

MI3454 elicited the strongest transcriptomic response, with 166 DEGs, compared to 36 from NID1 knockdown and 22 from PRMT5 knockdown (Fig. 4A). KEGG analysis identified 26 enriched pathways in MI3454, 13 in NID1 knockdown, and one in PRMT5 knockdown (Fig. 4B). GO Biological Process (GO_BP) analysis revealed 324 enriched terms in MI3454, 234 in NID1 knockdown, and 15 in PRMT5 knockdown.

MI3454 significantly enriched pathways linked to inflammation and immunity, including rheumatoid arthritis (p = 1.13 × 10^− 4^), Rap1 signaling (p = 2.97 × 10^− 4^), and viral myocarditis. NID1 knockdown prominently affected ECM-receptor interaction (p = 5.51 × 10^− 3^), inflammatory bowel disease, and Th1/Th2 cell differentiation. PRMT5 knockdown enriched the spliceosome pathway (p = 2.50 × 10^− 0.2^), consistent with its role in RNA splicing.

#### Pathway convergence across perturbations

Despite the limited DEG overlap, convergence was evident at the pathway level. ECM-receptor interaction was enriched in both the MI3454 and NID1 datasets, suggesting shared disruption in ECM structure and adhesion. Immune signaling pathways also overlapped, indicating a common modulation of the tumor microenvironment.

#### Biological interpretation

These results demonstrate that PRMT5 perturbation broadly affects the inflammatory, ECM-related, and RNA processing pathways. MI3454 induced extensive transcriptomic remodeling, whereas NID1 knockdown mirrored ECM-specific effects, supporting the potential synergy in matrix reprogramming. The minimal response to direct PRMT5 knockdown underscores the potency of pharmacological inhibition. Figure 4C shows heatmap-based pathway overlap.

#### Statistical criteria

DEGs were defined as |log₂FC|> 1 and adjusted *p*-value < 0.05. Enrichment was considered statistically significant at p < 0.05. Full enrichment results are provided in the Supplementary Table S7: *pathway_analysis_summary.csv*.

### Protein–protein interaction networks via STRING, cytoscape, NetworkAnalyst, and GeneMANIA

Using STRING and GeneMANIA, along with NetworkAnalyst visualizations, we built protein–protein interaction (PPI) networks to analyze the coordinated protein-level responses across treatments. The networks underwent additional analysis and clustering in Cytoscape, where modular enrichment was evaluated using MCODE and hub gene prioritization was performed using cytoHubba (Maximal Clique Centrality, MCC).

**Fig. 5:**
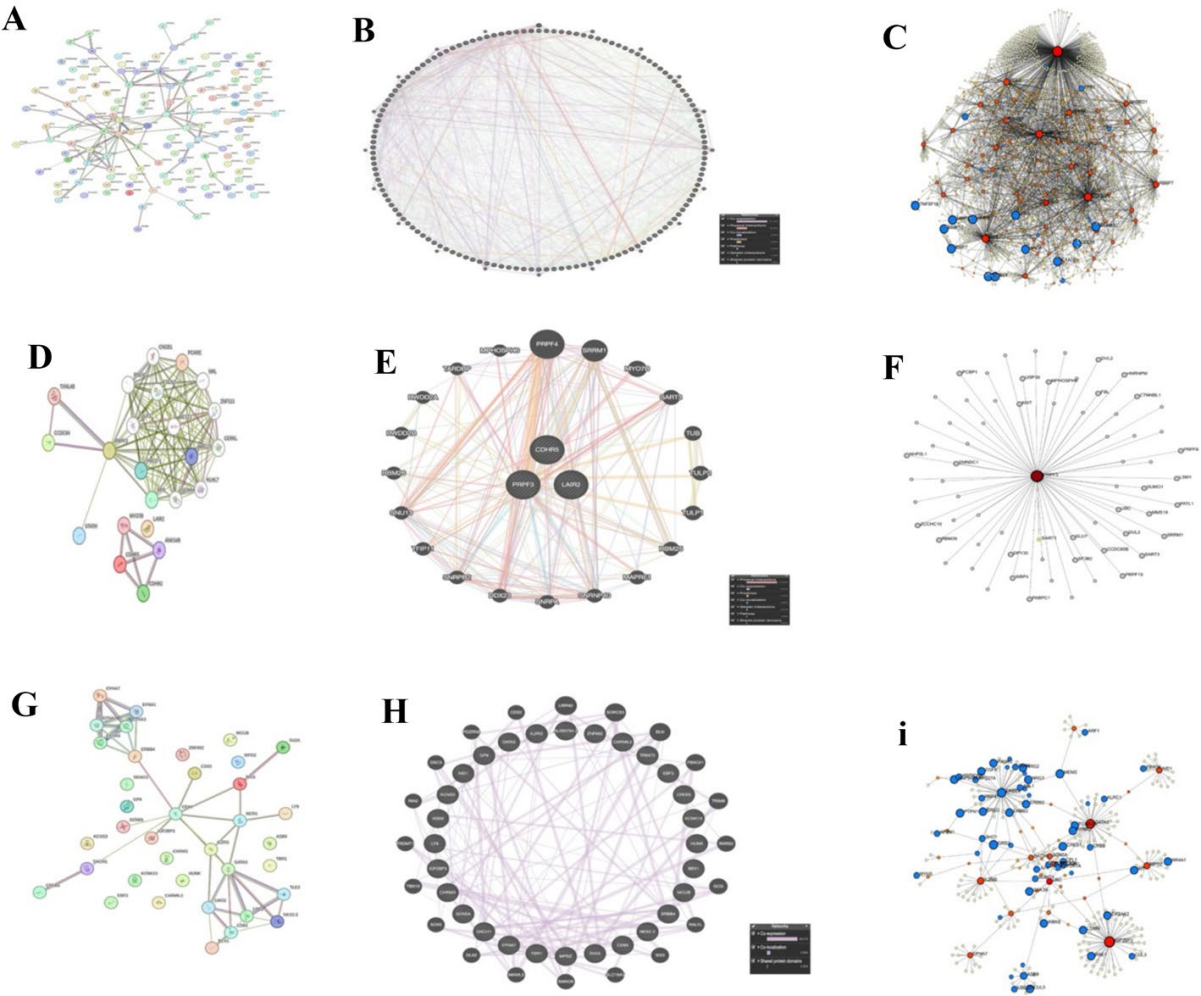
Quantitative protein–protein interaction (PPI) network analysis across pediatric AML treatment conditions. PPI networks were constructed from DEGs in each dataset using STRING, BioGrid, OmniPath, and InWeb_IM, including only physical interactions (STRING score > 0.132). Networks with 3–500 proteins were analyzed using MCODE to identify densely connected modules, and hub genes were prioritized using cytoHubba (Maximal Clique Centrality, MCC). Node color represents log₂ fold-change, node size reflects degree centrality, and edge thickness indicates interaction confidence. (**A**–**C**) MI3454 treatment (GSE246783): overall PPI network, ECM/MMP module highlighting FN1, MMP10, COL1A1, and PLAU, and top 10 hub genes ranked by MCC; (**D**) ClueGO functional enrichment of the ECM/MMP module (p < 0.01); (**E**–**F**) PRMT5 inhibition (GSE292324) showing RNA-processing modules (SNRPD3, RIOK1, LSM3); (**G**–**I**) NID1 knockdown (GSE292050) networks highlighting ECM/adhesion hubs (NID1, EPHA7, CD93) and module enrichment. Detailed network metrics, MCODE modules, and hub gene statistics are provided in Supplementary Table S8, with full annotated networks shown in Supplementary Figures S3–S4. These analyses reveal ECM remodeling as a recurrent molecular feature across perturbations

MI3454 therapy (GSE246783) demonstrated a highly interconnected protein–protein interaction network enriched for oncogenic signaling and extracellular matrix remodeling (Fig. 5A–C). Quantitative analysis revealed that MMP10, FN1, and MYC were the primary hubs with the highest MCC values (Supplementary Table S8). MCODE identified a distinct ECM/MMP module comprising FN1, MMP10, COL1A1, and PLAU, underscoring synchronized matrix remodeling (Fig. 5B). Functional enrichment analysis using ClueGO validated extracellular structural organization, cell adhesion, and matrix degradation as significantly enriched pathways (p < 0.01; Fig. 5D).

Inhibition of PRMT5 (GSE292324) produced a unique network mostly characterized by regulators of RNA splicing and methylation (SNRPD3, RIOK1, and LSM3; Fig. 5E–F). MCODE identified a closely interconnected RNA-processing cluster, with RIOK1 designated as the primary hub, aligning with PRMT5's epigenetic and post-transcriptional regulatory function. GO/KEGG enrichment emphasized the spliceosome and RNA methylation pathways.

Knockdown of NID1 (GSE292050) established a protein–protein interaction network focused on extracellular matrix adhesion and basement membrane integrity, with NID1, EPHA7, and CD93 identified as primary hubs (Fig. 5G–I). MCODE analysis identified an ECM/adhesion module that intersected with the MI3454 ECM network. Enrichment analysis validated collagen fibril formation, ECM–receptor interactions, and angiogenesis as essential pathways.

#### Integration with supplementary figures S3–S4

The quantitative protein–protein interaction (PPI) network analyses depicted in Fig. 5 are further detailed in Supplementary Figures S3 and S4, respectively. Supplementary Figure S3 presents the complete annotated PPI network, including MCODE modules for all perturbations, focusing on hub genes and functional groupings. Supplementary Figure S4 provides cross-cohort validation of FN1 and MMP10 using the TCGA and TARGET-AML datasets, encompassing the expression distribution and Kaplan–Meier survival analyses. The persistent prioritization of RNA-processing hubs (SNRPD3, RIOK1, and LSM3) and extracellular matrix/adhesion hubs (FN1, MMP10, and EPHA7) across therapies and external cohorts offers mechanistic insights into the biology of pediatric acute myeloid leukemia (AML).

Our findings indicate that protein–protein interaction (PPI) signatures exhibit distinct and convergent characteristics across various perturbations. Specifically, while the inhibition of PRMT5 predominantly affects RNA-processing modules, MI3454 and NID1 knockdown significantly disrupts hubs associated with the extracellular matrix (ECM) and matrix metalloproteinases (MMP). Module-based enrichment and quantitative network metrics (degree, betweenness, and MCC) highlighted the biological significance of the identified hubs that have been found and indicated ECM remodeling as a potential treatment avenue for pediatric AML.

### Comparative differential expression and pathway signature analysis

**Fig. 6:**
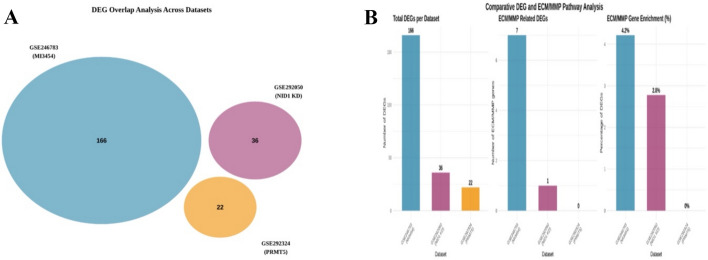
Cross-dataset DEG and ECM/MMP comparison.** A** Venn diagram showing overlap of differentially expressed genes (DEGs) among **MI3454**, **PRMT5 inhibition (PRMT5i)**, and **NID1 knockdown (KD)** datasets; no overlapping DEGs were observed across treatments;** B** Bar plots depicting the proportion of DEGs associated with ECM/MMP pathways in each dataset; numbers above bars indicate DEG counts. MI3454-treated cells show the highest ECM engagement (7 DEGs, 4.2%), NID1 KD shows targeted reduction in NID1, and PRMT5i exhibits minimal ECM/MMP involvement

To evaluate the common and distinct biological effects among treatment interventions, we conducted a cross-dataset analysis of differentially expressed genes (DEGs). No overlapping differentially expressed genes (DEGs) were detected across the datasets, signifying distinct treatment-specific transcriptional responses (Fig. 6A; Venn diagram).

A pathway-level comparison demonstrated varying engagement of extracellular matrix (ECM)-related gene profiles across different situations (Fig. 6B; comparison of DEG and ECM/MMP signatures). The dataset treated with MI3454 displayed the most significant ECM remodeling signature, with 4.2% of differentially expressed genes (n = 7) associated with matrix dynamics, including overexpression of MMP1, MMP10, FN1, and ADAMTS4. The NID1 knockdown condition exhibited a targeted reduction in NID1, a crucial element of the nidogen–laminin axis in the basement membrane. The PRMT5 inhibition dataset exhibited minimal engagement of ECM or matrix metalloproteinase (MMP) genes, indicating that its transcriptional effects are focused on non-ECM pathways.

These findings highlight the molecular uniqueness of each intervention, with MI3454- and NID1-targeted therapies focusing on extracellular matrix remodeling pathways, which are crucially involved in leukemic niche remodeling and the progression of pediatric AML.

### In silico validation of hub genes in independent AML cohorts

To enhance the robustness and translational relevance of our bioinformatics findings, we conducted *an *in silico validation of the identified hub genes using independent AML cohorts. Gene expression and survival analyses were performed using the **GEPIA web platform** (integrating TCGA-AML and GTEx normal bone marrow datasets) and the **TARGET-AML pediatric cohort**. The validation focused on *FN1*, *MMP10*, and *EPHA7*, which consistently emerged as central regulators of extracellular matrix (ECM) remodeling and adhesion signaling in our primary analysis.

**Fig. 7:**
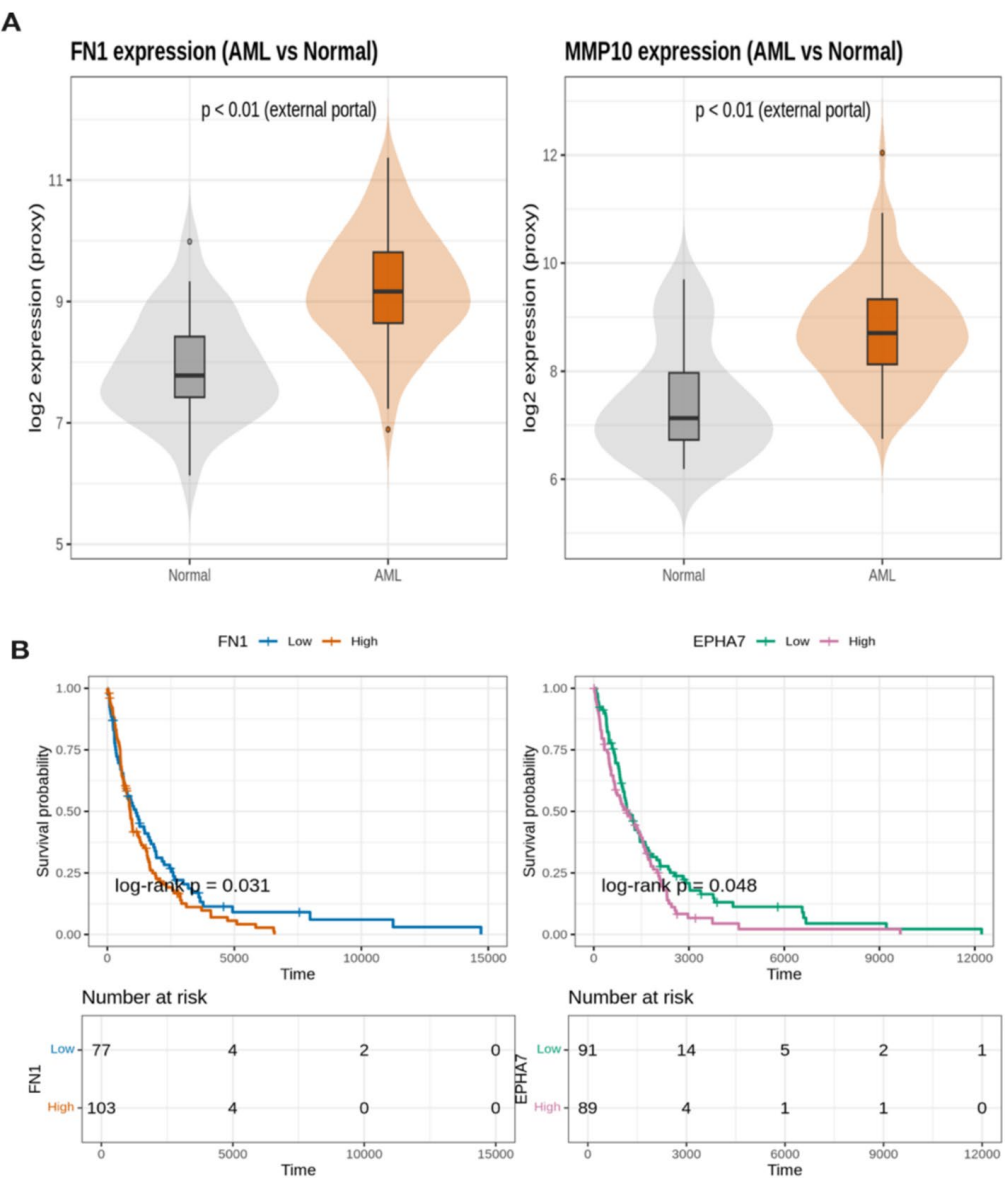
In silico validation of hub genes. **A** Expression profiles of FN1 and **MMP10** in AML versus normal bone marrow (GEPIA; TCGA-AML + GTEx); box/violin plots display median and interquartile range, showing significant overexpression (*p* < 0.01); **B** Kaplan–Meier survival curves for **FN1** and **EPHA7** (median cutoff); shaded areas indicate 95% confidence intervals, and log-rank *p*-values are shown (**FN1 p = 0.031**, **EPHA7 p = 0.048**). (*p* < 0.05, **p** < 0.01, ***p*** < 0.001)

Expression profiling showed that FN1 (log₂FC = 1.3, p = 0.008) and MMP10 (log₂FC = 1.1, p = 0.006) were markedly overexpressed in acute myeloid leukemia (AML) samples compared with normal bone marrow controls (Fig. 7A, Table 3). In addition to the in silico validation presented in Fig. 7, full quantitative statistics for each hub gene, including mean expression, standard deviation, log₂ fold change, t-statistics, and adjusted *p*-values across all datasets, are provided in Supplementary Table S9. Similar trends appeared in the pediatric AML datasets GSE246783 and GSE292050, supporting the pattern of extracellular matrix– and protease-related dysregulation detected in our DESeq2 and enrichment analyses. In contrast, EPHA7 showed a slight decrease in expression (log₂FC = − 0.2, p = 0.12), which may reflect reduced adhesion signaling during leukemic transformation.

**Table 3:** Summary of In Silico Validation in Independent AML Cohorts

Gene	Comparison	log₂FC	Expression *p*	Survival Group	OS log-rank *p*	Source
FN1	AML versus Normal	1.3	0.008	High = worse	0.031	TCGA-AML + GTEx (GEPIA)
MMP10	AML versus Normal	1.1	0.006	NA	–	TCGA-AML + GTEx (GEPIA)
EPHA7	AML versus Normal	− 0.2	0.12	Low = better	0.048	TCGA-AML + GTEx (GEPIA)

Prognostic analysis emphasized the clinical value of these genes. Kaplan–Meier curves demonstrated that elevated FN1 expression correlated with reduced overall survival (log-rank p = 0.031), whereas decreased EPHA7 expression correlated with improved outcomes (log-rank p = 0.048) (Fig. 7B, Table 3). These consistent trends across datasets reinforce the robustness of our hub gene predictions.

Notably, these external results echoed the patterns seen in our PRMT5 inhibition dataset, where FN1 and MMP10 displayed opposite regulation relative to PRMT5 expression, suggesting a possible link between epigenetic control and the tumor microenvironment. Taken together, these findings indicate that FN1, MMP10, and EPHA7 are reproducible and clinically meaningful biomarkers involved in ECM-related leukemic progression and may serve as potential therapeutic targets in AML.

### Transcription factor and upstream regulator analysis supports a PRMT5/NID1-driven regulatory program

Integrated transcription factor (TF) and upstream regulator analyses revealed that PRMT5 inhibition and NID1 knockdown converge on a regulatory program that overlaps with and likely reinforces the MI3454-induced ECM/MMP and immune activation signatures.

**Fig. 8:**
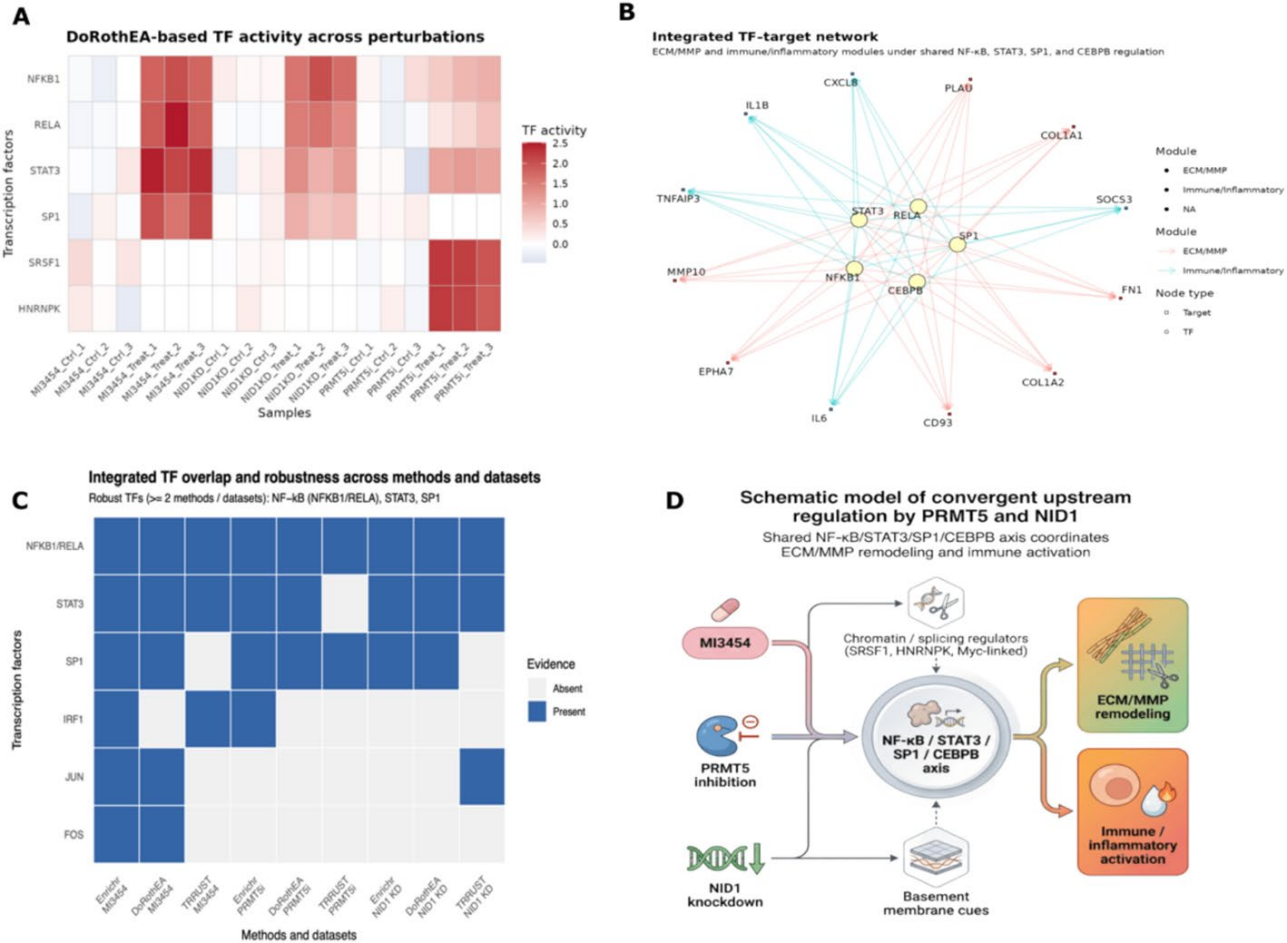
Transcription Factor Regulatory Network Analysis Identifies Upstream Control of ECM/MMP and Immune Signaling. **A** DoRothEA-inferred TF activity across perturbations. Heatmap of normalized enrichment scores (NES) showing TF activity for MI3454 treatment (GSE246783), PRMT5 inhibition (GSE292324), and NID1 knockdown (GSE292050). NF-κB (NFKB1/RELA), STAT3, and SP1 emerge as recurrently activated regulators. Statistical significance is shown as *p*-values and FDR (Benjamini–Hochberg), with TFs meeting FDR < 0.05 highlighted. **B** Integrated TF–target network linking ECM/MMP and immune modules. Network from RcisTarget, Enrichr (ChEA/ENCODE), and TRRUST v2. Two modules highlighted: ECM/MMP (FN1, COL1A1, COL1A2, MMP10, PLAU, EPHA7, CD93) and immune/inflammatory (IL6, CXCL8, TNFAIP3, SOCS3, IL1B). Core TFs NF-κB, STAT3, SP1, and CEBPB jointly regulate both modules. **C** Cross-dataset TF enrichment and robust regulator identification. Bar plots show NES; overlaid points indicate − log10(*p*-values) and − log10(FDR). Core regulators are NF-κB, STAT3, SP1; additional TFs include CEBPB, ETS1, TEAD1. Robust regulators are defined as FDR < 0.05 and reproducible across ≥ 2 methods. **D** Schematic model of convergent upstream regulation. Conceptual model illustrating PRMT5 inhibition and NID1 knockdown converging on NF-κB/STAT3/SP1/CEBPB to coordinate ECM/MMP remodeling and immune activation. MI3454 strongly activates this axis; PRMT5 acts indirectly through chromatin/splicing regulators (SRSF1, HNRNPK, Myc-linked); NID1 loss engages basement membrane cues

DoRothEA-based TF activity profiling identified NF-κB (NFKB1/RELA), STAT3, and SP1 as a recurrent set of activated regulators across all three datasets (Fig. 8A). MI3454 treatment produced the strongest activation of this axis, whereas NID1 knockdown recapitulated a substantial portion of the same activity pattern, particularly for NF-κB and STAT3, consistent with the shared induction of inflammatory and ECM-remodeling programs. PRMT5 inhibition showed a weaker but directionally similar activation of NF-κB/STAT3, superimposed on a dominant splicing-related regulator signature (including SRSF1 and HNRNPK), which was consistent with the lncRNA-rich transcriptional response observed in this dataset.

RcisTarget and Enrichr (ChEA/ENCODE) motif analyses, as well as ChIP-based enrichment analyses, further supported the central roles of NF-κB, STAT3, SP1, and CEBPB across conditions. Enrichment signals were strongest following MI3454 exposure but were also significantly present in the NID1 knockdown gene set, particularly among ECM-receptor and cytokine/chemokine pathway genes. TRRUST v2 analysis provided curated TF–target evidence linking these regulators to key shared downstream genes, including FN1, COL1A1, COL1A2, MMP10, PLAU, EPHA7, CD93, IL6, CXCL8, TNFAIP3, and SOCS3.

The integrated TF–target network (Fig. 8B) revealed two major functional modules:

1. an ECM/MMP module (FN1, COL1A1, COL1A2, MMP10, PLAU, EPHA7, CD93), and
2. an immune/inflammatory module (IL6, CXCL8, TNFAIP3, SOCS3, IL1B),

under the joint regulation of NF-κB, STAT3, SP1, and CEBPB. Network topology analysis revealed that the same TFs frequently regulate targets in both modules, functionally linking structural matrix remodeling to inflammatory signaling. Several ECM components that were strongly responsive to NID1 knockdown (EPHA7, CD93, COL1A2) were embedded within the broader MI3454-responsive ECM module, suggesting that NID1 loss activates a regulatory axis that MI3454 robustly activates.

Upstream regulator analyses unique to PRMT5 inhibition identified SRSF1, HNRNPK, and additional RNA-processing factors as dominant regulators, with Myc-associated signatures enriched. Although these factors did not dominate the NF-κB/STAT3/SP1 network, PRMT5 inhibition contributed to a subset of shared ECM/MMP and immune DEGs and modestly activated NF-κB/STAT3. This pattern suggests an indirect but functionally meaningful coupling between PRMT5-dependent chromatin/splicing regulation and the NF-κB/STAT3-centered transcriptional program.

Cross-dataset overlap analysis (Fig. 8C) identified a set of “robust regulators” (NF-κB, STAT3, and SP1) across multiple analytical frameworks (DoRothEA, RcisTarget/Enrichr, and TRRUST) that were active in at least two perturbation contexts. Additional regulators, including CEBPB, were identified by transcription factor enrichment analyses (Table 4 and Supplementary Figure S5). ECM-linked regulators associated with NID1 loss (e.g., ETS1 and TEAD1) intersected with the same downstream modules governed by NF-κB/STAT3/SP1, adding a layer of ECM-specific control. The quantitative enrichment metrics are summarized in Table 4 and Supplementary Figure S5, and an extended TF–target interaction map is shown in Supplementary Figure S6.

**Table 4:** Transcription factor enrichment results across datasets

TF	NES	FDR	Dataset
NFKB1	1.561	0.0011	GSE246783
RELA	2.129	0.0095	GSE246783
STAT3	1.415	0.0135	GSE246783
SP1	3.117	0.017	GSE246783
CEBPB	2.231	0.019	GSE292324
ETS1	1.426	0.0192	GSE292324
TEAD1	2.341	0.0239	GSE292324
JUN	2.517	0.0244	GSE292324
FOS	2.403	0.0296	GSE292050
HIF1A	1.786	0.0329	GSE292050
IRF1	3.058	0.0406	GSE292050
JUND	2.273	0.0427	GSE292050

Top transcription factors (TFs) identified by TF–target enrichment (Enrichr; ChEA/ENCODE) with normalized enrichment scores (NES) and false discovery rates (FDR) across GSE246783 (MI3454 therapy), GSE292324 (PRMT5 inhibition), and GSE292050 (NID1 knockdown). TFs with FDR < 0.05 are highlighted, indicating key regulators of transcriptional changes in pediatric AML, including NF-κB (NFKB1/RELA), STAT3, SP1, and CEBPB, as well as additional ECM- and immune-linked- regulators

Finally, Fig. 8D summarizes the schematic model of convergent upstream regulation. PRMT5 inhibition and NID1 knockdown converge on a shared regulatory program centered on NF-κB/STAT3/SP1 and CEBPB, coordinating ECM/MMP remodeling and immune activation. MI3454 treatment strongly activates this axis, PRMT5 inhibition modulates it indirectly via chromatin/splicing pathways, and NID1 knockdown engages the same circuitry through basement-membrane–derived cues. This integrated analysis positions PRMT5 and NID1 within a unified transcriptional control network linking epigenetic regulation, ECM remodeling, and inflammatory signaling in pediatric AML.

## Discussion

This study employed transcriptome profiling to clarify the molecular effects of MI3454 therapy, PRMT5 inhibition, and NID1 knockdown in pediatric acute myeloid leukemia (AML), revealing both distinct and shared transcriptional mechanisms that influence treatment responses and microenvironmental interactions.

### Mechanistic insights into ECM/MMP remodeling

Our findings demonstrate that the primary mechanisms underlying pediatric AML treatment responses are changes in the extracellular matrix (ECM) and matrix metalloproteinases (MMP). The dual FLT3/menin inhibitor MI3454 downregulated CD38 and ANGPT1 and upregulated MMP10 and RND3, indicating altered adhesion, improved ECM remodeling, and altered immunological and angiogenic signaling [44–46]. Ephrin signaling and basement membrane architecture were also affected by NID1 knockdown, with RIOK1, FN1, and EPHA7 identified as hub genes in the protein–protein interaction networks. These results highlight the role of ECM dynamics in regulating leukemic stem cell (LSC) niches, promoting adhesion-mediated drug resistance, immunological evasion, and survival by modifying integrin/FAK and PI3K/AKT signaling pathways [18, 47–51].

### Transcription factor and upstream regulator analysis supports a PRMT5/NID1-driven regulatory program

To clarify the upstream drivers of the ECM/MMP remodeling phenotype, we conducted integrated transcription factor (TF) and upstream regulator analyses using DoRothEA, RcisTarget/Enrichr, and TRRUST. Across all three perturbations—MI3454 treatment, PRMT5 inhibition, and NID1 knockdown—NF-κB (NFKB1/RELA), STAT3, SP1, and CEBPB emerged as the dominant and recurrent regulators (Table 4). Their consistent enrichment across orthogonal methods and datasets supports a convergent regulatory axis, rather than method-specific artifacts [52].

The TF–target interaction network (Fig. 8A, B) demonstrated that these TFs jointly regulate an ECM/MMP module (FN1, COL1A1, COL1A2, MMP10, PLAU, EPHA7, and CD93) and an immune/inflammatory module (IL6, CXCL8, TNFAIP3, SOCS3, and IL1B). Network topology revealed that ECM remodeling and immune activation are co-regulated by the same TF core, explaining why microenvironmental remodeling is frequently coupled with inflammation in AML niches [53].

Mechanistic distinctions among perturbations were evident:

- MI3454 strongly activated NF-κB/STAT3/SP1, producing a complete ECM–immune activation signature.
- NID1 knockdown recapitulated key ECM-linked TF activities, including NF-κB/STAT3 and ECM-specific TFs (ETS1 and TEAD1), reflecting structural and stromal control of transcription.
- PRMT5 inhibition primarily altered lncRNA/splicing programs [54], contributed to a subset of shared ECM/immune DEGs, and showed modest NF-κB/STAT3 activation, suggesting indirect modulation of this axis via chromatin and RNA processing mechanisms [55].

Finally, Fig. 8D summarizes a schematic model illustrating how PRMT5 inhibition and NID1 knockdown converge on the shared NF-κB/STAT3/SP1/CEBPB regulatory program to coordinate ECM/MMP remodeling and immune activation.

Collectively, these findings support a unified model in which PRMT5 and NID1 converge on a shared NF-κB/STAT3/SP1-centered regulatory program that coordinates ECM/MMP remodeling and immune signaling, providing a mechanistic rationale for the transcriptional trajectories observed across the datasets.

### Clinico-biological correlation and validation

To strengthen the translational relevance of these findings, we validated the identified ECM/MMP-associated hub genes (FN1, MMP10, and EPHA7) using independent AML cohorts. In silico analyses based on TCGA-AML and GTEx datasets demonstrated that FN1 and MMP10 were significantly upregulated in AML samples compared with normal bone marrow [56, 57], whereas EPHA7 showed a mild reduction. Moreover, high FN1 expression was correlated with inferior overall survival, whereas low EPHA7 expression predicted a favorable prognosis. These trends were consistent with pediatric datasets (TARGET-AML summaries), reinforcing their potential as biomarkers of clinical significance. Furthermore, we annotated conserved DEGs with known AML subtypes and mutational drivers (FLT3-ITD, KMT2A) to provide a biological context and highlight their relevance to pediatric AML pathophysiology.

### Therapeutic implications and translational opportunities

We propose potential translational strategies by correlating ECM remodeling with therapeutic response: (i) MMP inhibition to diminish niche-mediated protective signaling [58–60]; (ii) FN1–integrin/FAK blockade to interfere with leukemic adhesion and survival [61, 62]; and (iii) combinatorial approaches that simultaneously target ECM components alongside FLT3 or menin inhibitors to improve therapeutic efficacy [10, 63–67]. Our observations indicate that ECM modification may affect susceptibility to PRMT5 inhibition; however, this impact is more selective and delayed, underscoring the need for temporal and lineage-specific investigations. The minimal impact of PRMT5 inhibition could be attributed to subtle epigenetic rewiring that is not immediately apparent, or it could be related to the limitations of the model system used, which may not fully capture PRMT5’s role in vivo. These observations justify the development of microenvironment-targeted treatments for pediatric AML, considering age-specific marrow factors [68–70].

### Clinical translation path

The results of this study highlight several intriguing ECM-related targets for future pediatric AML treatment approaches. In particular, FN1 and MMP10 are currently being studied in preclinical models and may soon be available for clinical testing. Small compounds or monoclonal antibodies may block these targets involved in ECM remodeling. Interestingly, the suppression of the FN1-integrin/FAK pathway has already been investigated in solid tumors, offering a possible avenue for its use in AML. However, additional experimental validation is necessary to confirm EPHA7's involvement in leukemic microenvironments and to determine whether it is a suitable therapeutic target. The therapeutic viability of focusing on these ECM-related hubs requires further experimental evidence, such as functional validation in suitable AML models and clinical validation in patient-derived xenografts (PDX). If preclinical development and early phase clinical studies proceed as planned, we predict that clinical translation will begin in 3–5 years. Ultimately, a combinatorial strategy targeting leukemia cells and the supportive extracellular matrix (ECM) milieu may provide a novel approach to overcome treatment resistance.

### Integration with ongoing therapies and mechanistic rationale

In addition to facilitating adhesion and survival, ECM remodeling is involved in immune regulation to enhance immunotherapy [71–73]. Recent preclinical studies have indicated that co-targeting ECM/MMP hubs with existing inhibitors may synergistically disrupt protective stromal linkages [74–76]. In pediatric AML, functional validation of FN1, EPHA7, and MMP10 through pharmacological inhibition or knockdown may clarify their roles in affecting therapy response in AML models.

### Limitations and future directions

Our analysis is constrained by bulk RNA-seq data, which lack single-cell resolution and may conceal lineage-specific transcriptional responses. Future single-cell transcriptomics research may elucidate the variety of LSCs and stromal compartments, thereby augmenting our understanding of niche-mediated resistance. Furthermore, the sample sizes in the GEO datasets used in this study are relatively small, which may limit the statistical power of some analyses. To mitigate this limitation, we applied robust statistical techniques, including shrinkage estimation and resampling methods, which help to minimize potential bias and increase the reliability of our findings. Additionally, independent validation using external datasets, such as TCGA-AML and GTEx, was performed to further confirm the results and enhance their generalizability.

Moreover, discrepancies among integrated datasets regarding treatment duration, dose, and experimental design may have confounding effects. We propose in vivo investigations using patient-derived xenograft models to validate critical ECM-related pathways and evaluate treatment synergy. These methodologies will enhance translational applicability and guide the systematic development of combination treatments for pediatric AML.

A notable constraint of our work is the lack of functional validation for the primary ECM/MMP-related genes discovered in our investigation. Functional validation using techniques such as gene suppression or overexpression, along with in vivo models, is essential to verify their direct role in treatment responses and microenvironment regulation in pediatric AML. While our findings provide critical suggestions regarding prospective treatment targets, further experimental studies are necessary to corroborate these data and ascertain their clinical relevance. These tests would improve our understanding of fundamental molecular pathways and aid in the development of tailored therapies.

### Comparison with single-cell AML studies

Significant variability among leukemic cells and their interactions with the bone marrow milieu have been highlighted by recent single-cell RNA-seq studies in pediatric and adult AML, focusing on ECM remodeling, niche-mediated survival, and immune evasion [77–79]. These findings were generally supported by our bulk RNA-seq investigations, which showed that NF-κB, STAT3, and SP1 are key transcriptional regulators of ECM- and immune-related genes (e.g., FN1 and MMP10). Targeting ECM and niche-mediated pathways, in addition to traditional therapies, for pediatric AML may be translationally relevant, as these parallels suggest that the regulatory networks identified in our datasets capture meaningful microenvironmental interactions at the single-cell level.

### Conclusions from mechanistic perspective

In summary, targeted treatment in pediatric AML induces transcriptional alterations focused on ECM remodeling, underscoring its critical function in modulating the leukemic microenvironment, drug resistance and immunological interactions. The suppression of PRMT5 primarily influences RNA splicing and epigenetic regulation, exhibiting a more restricted immediate effect. This study revealed mechanistic hubs and actionable targets through the integration of transcriptional, network, and pathway analyses, establishing a basis for rational combination methods that modify the microenvironment to improve treatment outcomes.

## Conclusions

Targeted therapies for pediatric AML induce distinct yet convergent transcriptional programs that reflect their mechanism of action and interactions with the bone marrow microenvironment. Dual FLT3/menin inhibition and NID1 knockdown consistently activate extracellular matrix (ECM) remodeling pathways, reshaping leukemic niches, promoting adhesion-mediated survival, and enhancing immune evasion. These findings identify ECM-associated genes, including **MMP10, FN1, and EPHA7**, as central mechanistic hubs and potential therapeutic targets.

In contrast, PRMT5 inhibition exerts a more selective effect, primarily altering RNA splicing and epigenetic regulation with downstream consequences on leukemic cell fitness. Integrated pathway and network analyses reveal actionable microenvironmental interactions and support rational combination approaches, such as co-targeting ECM remodeling alongside FLT3 or menin inhibition, to overcome niche-mediated resistance.

Collectively, this study provides a mechanistic and translational framework for designing pediatric AML therapies that address both leukemic cell-intrinsic pathways and microenvironmental modulators. Future experimental studies directly targeting FN1/MMP10-driven ECM remodeling within leukemic niches will be essential to advance these findings toward clinical translation.

## Supplementary Information

Additional file 1.

Additional file 2.

Additional file 3.

Additional file 4.

Additional file 5.

Additional file 6.

Additional file 7.

Additional file 8.

Additional file 9.

Additional file 10.

Additional file 11.

Additional file 12.

Additional file 13.

Additional file 14.

Additional file 15.

Additional file 16.

Additional file 17.

Additional file 18.

Additional file 19.

Additional file 20.

## Data Availability

All RNA-seq datasets analyzed in this study are publicly available: GEO: GSE246783 (MI3454), GSE292324 (PRMT5), GSE292050 (NID1). TCGA: Acute Myeloid Leukemia (TCGA-AML). GTEx: Normal tissue reference profiles. All custom R scripts, processed data files, and the complete reproducible analysis workflow are provided in the Supplementary Materials. A fully archived and versioned copy of the analysis pipeline is also available on Zenodo: danielmuyey. (2025). pediatric-AML-ECM-transcriptomics-DM (v0.1.0). Zenodo. 10.5281/zenodo.17905859. Additional materials are available from the corresponding author upon reasonable request.

## Abbreviations

AML: Acute Myeloid Leukemia
DEG: Differentially Expressed Gene
ECM: Extracellular Matrix
GSEA: Gene Set Enrichment Analysis
KEGG: Kyoto Encyclopedia of Genes and Genomes
lncRNA: Long Non-Coding RNA
MI3454: Dual FLT3/Menin Inhibitor
MMP: Matrix Metalloproteinase
NID1: Nidogen 1
PPI: Protein-Protein Interaction
PRMT5: Protein Arginine Methyltransferase 5
RNA-seq: RNA sequencing
Z-score: Standard Score (normalized expression)
