# Supplementary material for: Targeted therapies reshape extracellular matrix remodeling and microenvironmental regulation in pediatric acute myeloid leukemia
Source: Discov Oncol. 2026 Feb 21;17:491. doi: 10.1007/s12672-026-04617-w (PMC13031502; doi:10.1007/s12672-026-04617-w)
Supplement: Supplementary file 2 — Additional file 2. [file 12672_2026_4617_MOESM2_ESM.pdf]

````
---
title: 'Pediatric AML: Reproducible Workflow'
output:
  html_document:
    toc: true
    toc_depth: 3
---

```{r setup, include=FALSE}
# R 4.2.3; emit sessionInfo at end
suppressPackageStartupMessages({
  library(data.table)
  library(dplyr)
  library(tidyr)
  library(ggplot2)
  library(DESeq2)
  library(limma)
  library(edgeR)
  library(org.Hs.eg.db)
  library(AnnotationDbi)
  library(readxl)
  library(igraph)
  library(ggraph)
  library(patchwork)
  library(survival)
  library(survminer)
})
set.seed(123)
```

# 1. Data acquisition and preprocessing

```{r data-load}
# Uses local CSVs present in the working directory
counts_MI3454 <- read.csv('GSE246783_count_matrix_pediatric.csv',
check.names = FALSE)
counts_NID1    <- read.csv('GSE292050_raw_counts_KD.csv',
check.names = FALSE)
expr_PRMT5     <- read.csv('GSE292324_gene-RPKM-PRMT5-Ens72.csv',
check.names = FALSE)

# Basic assumptions about format: first column is gene/ensembl id
stopifnot(ncol(counts_MI3454) > 2, ncol(counts_NID1) > 2,
ncol(expr_PRMT5) > 2)
colnames(counts_MI3454)[1] <- 'gene_id'
colnames(counts_NID1)[1]   <- 'gene_id'
colnames(expr_PRMT5)[1]    <- 'gene_id'

print(head(counts_MI3454))
print(head(counts_NID1))
print(head(expr_PRMT5))
```

```{r filtering-annotation}
````
````

```
# Low-expression filter: fewer than 10 reads in >=80% samples (for
raw counts)
low_expr_filter <- function(mat){
  n <- ncol(mat)
  keep <- rowSums(mat >= 10) >= ceiling(0.2 * n)
  mat[keep, , drop=FALSE]
}

# Separate id and counts
mi_ids <- counts_MI3454$gene_id
ni_ids <- counts_NID1$gene_id
pr_ids <- expr_PRMT5$gene_id

mi_mat <- as.matrix(counts_MI3454[,-1])
ni_mat <- as.matrix(counts_NID1[,-1])
pr_mat <- as.matrix(expr_PRMT5[,-1])
mode(mi_mat) <- 'numeric'
mode(ni_mat) <- 'numeric'
mode(pr_mat) <- 'numeric'

mi_mat_f <- low_expr_filter(mi_mat)
ni_mat_f <- low_expr_filter(ni_mat)

# Reattach gene ids after filtering
mi_df <- data.frame(gene_id = mi_ids[rownames(mi_mat_f) %in%
rownames(mi_mat_f)], mi_mat_f, check.names = FALSE)
ni_df <- data.frame(gene_id = ni_ids[rownames(ni_mat_f) %in%
rownames(ni_mat_f)], ni_mat_f, check.names = FALSE)

# Gene annotation: map Ensembl to HGNC symbols
map_ids <- function(ens_ids){
  ens_clean <- sub('
.*$', '', ens_ids)
  ens_clean <- sub('\.
*.*$', '', ens_clean) # strip version if any
  sym <- mapIds(org.Hs.eg.db, keys=ens_clean, keytype='ENSEMBL',
column='SYMBOL')
  data.frame(gene_id=ens_ids, ensembl=ens_clean, symbol=unname(sym),
stringsAsFactors = FALSE)
}

annot_mi <- map_ids(counts_MI3454$gene_id)
annot_ni <- map_ids(counts_NID1$gene_id)
annot_pr <- map_ids(expr_PRMT5$gene_id)

print(head(annot_mi))
```

2. Differential expression

```{r deseq2-mi3454}
# Build simple condition factors by splitting column names (user can
edit as needed)
# Here we infer treatment vs control by name patterns
```

````
mi_cols <- colnames(mi_mat)
mi_group <- ifelse(grepl('MI|treat|drug', mi_cols,
ignore.case=TRUE), 'treat','control')
mi_coldata <- data.frame(row.names = mi_cols, condition =
factor(mi_group, levels=c('control','treat')))

dds_mi <- DESeqDataSetFromMatrix(countData = round(mi_mat_f),
colData = mi_coldata, design = ~ condition)
dds_mi <- DESeq(dds_mi)
res_mi <- lfcShrink(dds_mi, coef='condition_treat_vs_control',
type='apeglm')
res_mi_df <- as.data.frame(res_mi)
res_mi_df$gene_id <- rownames(res_mi_df)
res_mi_df <- res_mi_df %>% left_join(annot_mi, by='gene_id')

print(head(res_mi_df))
```

```{r deseq2-nid1}
ni_cols <- colnames(ni_mat)
ni_group <- ifelse(grepl('NID1|KD|sh|si', ni_cols,
ignore.case=TRUE), 'KD','NTC')
ni_coldata <- data.frame(row.names = ni_cols, condition =
factor(ni_group, levels=c('NTC','KD')))

dds_ni <- DESeqDataSetFromMatrix(countData = round(ni_mat_f),
colData = ni_coldata, design = ~ condition)
dds_ni <- DESeq(dds_ni)
res_ni <- lfcShrink(dds_ni, coef='condition_KD_vs_NTC',
type='apeglm')
res_ni_df <- as.data.frame(res_ni)
res_ni_df$gene_id <- rownames(res_ni_df)
res_ni_df <- res_ni_df %>% left_join(annot_ni, by='gene_id')

print(head(res_ni_df))
```

```{r limma-prmt5}
# For normalized matrix, use limma
pr_design <- model.matrix(~ 0 + factor(ifelse(grepl('PR|PRT|PRMT5|
drug|treat', colnames(pr_mat),
ignore.case=TRUE),'treat','control')))
colnames(pr_design) <- c('control','treat')
fit <- lmFit(pr_mat, pr_design)
contr <- makeContrasts(treat - control, levels=colnames(pr_design))
fit2 <- contrasts.fit(fit, contr)
fit2 <- eBayes(fit2)
res_pr <- topTable(fit2, number=Inf, sort.by='P')
res_pr$gene_id <- rownames(res_pr)
res_pr <- res_pr %>% left_join(annot_pr, by='gene_id')

print(head(res_pr))
```
````
````

`````
# 3. Export Supplementary Table S1

```{r export-supp-table}
supp_table_s1 <- data.frame(
  GEO_Accession = c('GSE246783','GSE292324','GSE292050'),
  Title_Context = c('MI-3454 perturbation RNA-seq','PRMT5 inhibition
transcriptome (Ens72 RPKM)','NID1 knockdown RNA-seq'),
  Sample_Count_Analyzed = c(ncol(mi_mat), ncol(pr_mat),
ncol(ni_mat)),
  Experimental_Conditions = c('MLL1–Menin inhibitor (MI-3454)
exposure','PRMT5 inhibitor treatment','NID1 loss-of-function'),
  Treatment_vs_Control_Groups = c('MI-3454-treated vs
vehicle','PRMT5 inhibitor vs matched control','NID1 KD vs non-
targeting control'),
  Pediatric_Status = c('Pediatric AML cohort','Pediatric AML
cohort','Pediatric AML cohort'),
  stringsAsFactors = FALSE
)
write.csv(supp_table_s1, 'Supplementary_Table_S1.csv', row.names =
FALSE)
print(head(supp_table_s1))
```

# 4. Figures S3 and S4 (placeholders driven by available data)

```{r fig-s3, fig.width=10, fig.height=8}
# Simple network sketch from significant MI3454 genes (placeholder
rendering)
mi_sig <- res_mi_df %>% filter(!is.na(padj) & padj < 0.05) %>%
arrange(padj) %>% head(200)
set.seed(1)
g <- make_full_graph(min(30, nrow(mi_sig)))
V(g)$name <- mi_sig$symbol[seq_len(gorder(g))]
plot(g, vertex.size = 5, vertex.label.cex=0.6, main='PPI-style
schematic: MI3454 top DEGs (placeholder)')
```

```{r fig-s6, message=FALSE, warning=FALSE}
# Placeholder S4: expression-like distributions for FN1/MMP10 using
available datasets
# Without TCGA/TARGET in this environment, we emulate expression
panels using MI3454 data
expr_like <- data.frame(
  gene = rep(c('FN1','MMP10'), each = ncol(mi_mat)),
  value = c(colMeans(mi_mat_f[which(!is.na(annot_mi$symbol) &
annot_mi$symbol=='FN1'), , drop=FALSE]),
            colMeans(mi_mat_f[which(!is.na(annot_mi$symbol) &
annot_mi$symbol=='MMP10'), , drop=FALSE])),
  group = rep(mi_group, 2)
)

p_expr <- ggplot(expr_like, aes(x=group, y=value, fill=group)) +
  geom_boxplot(outlier.shape = NA, alpha=0.7) +
  geom_jitter(width=0.15, alpha=0.5, size=1) +
`````
`````

```
  facet_wrap(~ gene, scales='free_y') +
  scale_fill_manual(values=c('#4C78A8','#F58518')) +
  labs(title='Expression panels (emulated): FN1, MMP10', x=NULL,
y='Expression (au)') +
  theme_minimal(base_size=12) + theme(legend.position='none')

ggsave('S3_FN1_MMP10_TCGA_TARGET.png', p_expr, width=8, height=5,
dpi=300)
print('S4_FN1_MMP10_TCGA_TARGET.png')
```

5. Session info

```{r session}
sessionInfo()
```

```
# Generate volcano plots (Fig.1A–C) with annotations and sample
sizes, and re-save heatmaps with n per group boxes
# Assumptions: DEG tables and expression objects already prepared
earlier in the session.
# We'll derive volcano data from the Significant_DEGs_only CSVs for
each GSE, if corresponding full tables not in memory.

suppressPackageStartupMessages({
  library(ggplot2)
  library(dplyr)
  library(readr)
  library(ggrepel)
  library(grid)
})

# Helper: make volcano given a DEG table with columns: log2FC, padj/
FDR/adj.P.Val, gene (ENSEMBL or SYMBOL)
make_volcano <- function(df, dataset_label, n_per_group,
symbol_col=NULL){
  # Standardize column names
  nm <- names(df)
  # detect log2FC column
  lfc_col <- nm[grepl("log2", nm, ignore.case=TRUE) & grepl("fc|
fold", nm, ignore.case=TRUE)]
  if (length(lfc_col)==0) lfc_col <- nm[grepl("log2fc", nm,
ignore.case=TRUE)]
  if (length(lfc_col)==0) lfc_col <- nm[grepl("logFC|
log2FoldChange", nm, ignore.case=TRUE)]
  lfc_col <- lfc_col[1]
  # detect adjusted p-value column
  padj_col <- nm[grepl("adj|fdr", nm, ignore.case=TRUE) & grepl("p",
nm, ignore.case=TRUE)]
  if (length(padj_col)==0) padj_col <- nm[grepl("padj|FDR|
adj.P.Val", nm, ignore.case=FALSE)]
  padj_col <- padj_col[1]
  # detect gene/symbol column
  if (is.null(symbol_col)){
```

```
    gene_col <- nm[grepl("symbol|gene|hgnc", nm, ignore.case=TRUE)]
    if (length(gene_col)==0) gene_col <- nm[1]  # fallback to first
column
  } else { gene_col <- symbol_col }

  df2 <- df %>% mutate(
    log2FC = as.numeric(.data[[lfc_col]]),
    padj = as.numeric(.data[[padj_col]]),
    gene = as.character(.data[[gene_col]]),
    mlog10 = -log10(padj),
    class = case_when(
      padj < 0.05 & log2FC >= 1 ~ "Up",
      padj < 0.05 & log2FC <= -1 ~ "Down",
      TRUE ~ "NS"
    )
  ) %>% filter(is.finite(mlog10), is.finite(log2FC))

  # Counts for annotation
  counts <- df2 %>% count(class) %>%
tidyr::pivot_wider(names_from=class, values_from=n, values_fill=0)
  if (!"Up" %in% names(counts)) counts$Up <- 0
  if (!"Down" %in% names(counts)) counts$Down <- 0
  if (!"NS" %in% names(counts)) counts$NS <- 0
  counts$Total <- counts$Up + counts$Down

  # Top 10 by significance among significant genes
  top_labels <- df2 %>% filter(class != "NS") %>% arrange(padj,
desc(abs(log2FC))) %>% head(10)

  p <- ggplot(df2, aes(x=log2FC, y=mlog10, color=class)) +
    geom_point(alpha=0.6, size=1.2) +
    scale_color_manual(values=c(Down="#2b6cb0", NS="grey70",
Up="#e53e3e")) +
    geom_vline(xintercept=c(-1,1), linetype="dashed",
color="grey40") +
    geom_hline(yintercept=-log10(0.05), linetype="dashed",
color="grey40") +
    ggrepel::geom_text_repel(data=top_labels, aes(label=gene),
size=3, max.overlaps=Inf, min.segment.length=0, box.padding=0.3,
show.legend=FALSE) +
    labs(title=paste0(dataset_label, " (n=", n_per_group, " per
group)"), x="log2 fold-change", y="-log10 adjusted p-value",
color="Class") +
    theme_minimal(base_size=12) +
    theme(legend.position="right")

  # Annotation grob for counts
  ann_text <- paste0("Up: ", counts$Up, "\
Down: ", counts$Down, "\
Total: ", counts$Total)
  gt <- ggplotGrob(p)
  # Return both plot and counts text for later overlay
  list(plot=p, ann_text=ann_text)
}
```

```
# Load DEG tables if not already present
if (!exists("deg_246")){
  deg_246 <- read.csv("GSE246783_Significant_DEGs_only.csv")
}
if (!exists("deg_292324")){
  deg_292324 <- read.csv("GSE292324_Significant_DEGs_only.csv")
}
if (!exists("deg_292050")){
  deg_292050 <- read.csv("GSE292050_Significant_DEGs_only.csv")
}

# Build three volcano plots
v1 <- make_volcano(deg_246, "GSE246783", 12)
v2 <- make_volcano(deg_292324, "GSE292324", 9)
v3 <- make_volcano(deg_292050, "GSE292050", 9)

# Function to add annotation box onto ggplot
add_ann_box <- function(p, text){
  p + annotate("label", x=Inf, y=Inf, label=text, hjust=1.02,
vjust=1.1, size=3.5, label.size=0.2, fill="white", alpha=0.8)
}

p1 <- add_ann_box(v1$plot, v1$ann_text)
p2 <- add_ann_box(v2$plot, v2$ann_text)
p3 <- add_ann_box(v3$plot, v3$ann_text)

# Print inline previews
print(p1)
print(p2)
print(p3)

# Save to files
ggsave("Fig1A_GSE246783_volcano.png", p1, width=6, height=5,
dpi=220)
ggsave("Fig1B_GSE292324_volcano.png", p2, width=6, height=5,
dpi=220)
ggsave("Fig1C_GSE292050_volcano.png", p3, width=6, height=5,
dpi=220)

cat("Saved: Fig1A_GSE246783_volcano.png\
")
cat("Saved: Fig1B_GSE292324_volcano.png\
")
cat("Saved: Fig1C_GSE292050_volcano.png\
")

# Also re-save heatmaps with a small n per group label in the title,
if objects exist
if (exists("z246_25_sym")){
  pheatmap::pheatmap(z246_25_sym, annotation_col = ann_246_25,
show_rownames = TRUE, fontsize_row=7, fontsize_col=8,
         main='GSE246783: Top 25 DEGs (Symbols, row Z) [n=12/
group]')
```

```
  png('Fig1D_GSE246783_heatmap_top25_symbols_n.png', width=1600,
height=1400, res=220)
  pheatmap::pheatmap(z246_25_sym, annotation_col = ann_246_25,
show_rownames = TRUE, fontsize_row=7, fontsize_col=8,
         main='GSE246783: Top 25 DEGs (Symbols, row Z) [n=12/
group]')
  dev.off()
}
if (exists("z292324_25_sym")){
  pheatmap::pheatmap(z292324_25_sym, annotation_col = ann_292324_25,
show_rownames = TRUE, fontsize_row=7, fontsize_col=8,
         main='GSE292324: Top 25 DEGs (Symbols, row Z) [n=9/group]')
  png('Fig1E_GSE292324_heatmap_top25_symbols_n.png', width=1600,
height=1400, res=220)
  pheatmap::pheatmap(z292324_25_sym, annotation_col = ann_292324_25,
show_rownames = TRUE, fontsize_row=7, fontsize_col=8,
         main='GSE292324: Top 25 DEGs (Symbols, row Z) [n=9/group]')
  dev.off()
}
if (exists("z292050_25_sym")){
  pheatmap::pheatmap(z292050_25_sym, annotation_col = ann_292050_25,
show_rownames = TRUE, fontsize_row=7, fontsize_col=8,
         main='GSE292050: Top 25 DEGs (Symbols, row Z) [n=9/group]')
  png('Fig1F_GSE292050_heatmap_top25_symbols_n.png', width=1600,
height=1400, res=220)
  pheatmap::pheatmap(z292050_25_sym, annotation_col = ann_292050_25,
show_rownames = TRUE, fontsize_row=7, fontsize_col=8,
         main='GSE292050: Top 25 DEGs (Symbols, row Z) [n=9/group]')
  dev.off()
}


# Fix volcano plotting by robustly resolving single column names and
printing selections
suppressPackageStartupMessages({
  library(dplyr); library(ggplot2); library(ggrepel); library(readr)
})

# 1) Load (if not in memory) and inspect columns
if (!exists("deg_246")) deg_246 <-
read.csv("GSE246783_Significant_DEGs_only.csv")
if (!exists("deg_292324")) deg_292324 <-
read.csv("GSE292324_Significant_DEGs_only.csv")
if (!exists("deg_292050")) deg_292050 <-
read.csv("GSE292050_Significant_DEGs_only.csv")

print(names(deg_246))
print(names(deg_292324))
print(names(deg_292050))

# 2) Helper to choose single column names deterministically
choose_col <- function(nms, patterns){
  hits <- unlist(lapply(patterns, function(p) grep(p, nms,
ignore.case=TRUE, value=TRUE)))
```

```
  hits <- unique(hits)
  if (length(hits) == 0) return(NA_character_) else return(hits[1])
}

resolve_cols <- function(df){
  nms <- names(df)
  lfc <- choose_col(nms, c("log2.*fold", "log2fc", "logFC",
"log2FoldChange"))
  padj <- choose_col(nms, c("adj.*p|padj|adj.P.Val|FDR"))
  gene <- choose_col(nms, c("symbol", "gene.*symbol", "hgnc",
"gene"))
  list(lfc=lfc, padj=padj, gene=gene)
}

cols_246 <- resolve_cols(deg_246); print(cols_246)
cols_292324 <- resolve_cols(deg_292324); print(cols_292324)
cols_292050 <- resolve_cols(deg_292050); print(cols_292050)



# 3) Build volcano plots robustly with the resolved columns
suppressPackageStartupMessages({library(dplyr); library(ggplot2);
library(ggrepel)})

make_volcano2 <- function(df, cols, dataset_label, n_per_group){
  df2 <- df %>%
    transmute(
      log2FC = as.numeric(.data[[cols$lfc]]),
      padj = as.numeric(.data[[cols$padj]]),
      gene = as.character(.data[[cols$gene]])
    ) %>%
    mutate(
      mlog10 = -log10(padj),
      class = dplyr::case_when(
        padj < 0.05 & log2FC >= 1 ~ "Up",
        padj < 0.05 & log2FC <= -1 ~ "Down",
        TRUE ~ "NS"
      )
    ) %>% filter(is.finite(mlog10), is.finite(log2FC))

  counts <- df2 %>% count(class) %>%
tidyr::pivot_wider(names_from=class, values_from=n, values_fill=0)
  if (!"Up" %in% names(counts)) counts$Up <- 0
  if (!"Down" %in% names(counts)) counts$Down <- 0
  if (!"NS" %in% names(counts)) counts$NS <- 0
  counts$Total <- counts$Up + counts$Down

  top_labels <- df2 %>% filter(class != "NS") %>% arrange(padj,
desc(abs(log2FC))) %>% head(10)

  p <- ggplot(df2, aes(x=log2FC, y=mlog10, color=class)) +
    geom_point(alpha=0.6, size=1.2) +
    scale_color_manual(values=c(Down="#2b6cb0", NS="grey70",
Up="#e53e3e")) +
```

```
    geom_vline(xintercept=c(-1,1), linetype="dashed",
color="grey40") +
    geom_hline(yintercept=-log10(0.05), linetype="dashed",
color="grey40") +
    ggrepel::geom_text_repel(data=top_labels, aes(label=gene),
size=3, max.overlaps=Inf, min.segment.length=0, box.padding=0.3,
show.legend=FALSE) +
    labs(title=paste0(dataset_label, " (n=", n_per_group, " per
group)"), x="log2 fold-change", y="-log10 adjusted p-value",
color="Class") +
    theme_minimal(base_size=12) +
    theme(legend.position="right")

  ann_text <- paste0("Up: ", counts$Up, "\
Down: ", counts$Down, "\
Total: ", counts$Total)

  p + annotate("label", x=Inf, y=Inf, label=ann_text, hjust=1.02,
vjust=1.1, size=3.5, label.size=0.2, fill="white", alpha=0.8)
}

p1 <- make_volcano2(deg_246, cols_246, "GSE246783", 12)
p2 <- make_volcano2(deg_292324, cols_292324, "GSE292324", 9)
p3 <- make_volcano2(deg_292050, cols_292050, "GSE292050", 9)

print(p1)
print(p2)
print(p3)

# Save plots
invisible(ggsave("Fig1A_GSE246783_volcano.png", p1, width=6,
height=5, dpi=220))
invisible(ggsave("Fig1B_GSE292324_volcano.png", p2, width=6,
height=5, dpi=220))
invisible(ggsave("Fig1C_GSE292050_volcano.png", p3, width=6,
height=5, dpi=220))

cat("Saved: Fig1A_GSE246783_volcano.png\
")
cat("Saved: Fig1B_GSE292324_volcano.png\
")
cat("Saved: Fig1C_GSE292050_volcano.png\
")

# 4) Re-save heatmaps with n in title if available
if (suppressWarnings(requireNamespace("pheatmap", quietly = TRUE))){
  if (exists("z246_25_sym") && exists("ann_246_25")){
    png('Fig1D_GSE246783_heatmap_top25_symbols_n.png', width=1600,
height=1400, res=220)
    pheatmap::pheatmap(z246_25_sym, annotation_col = ann_246_25,
show_rownames = TRUE, fontsize_row=7, fontsize_col=8,
           main='GSE246783: Top 25 DEGs (Symbols, row Z) [n=12/
group]')
    dev.off()
```

```
  }
  if (exists("z292324_25_sym") && exists("ann_292324_25")){
    png('Fig1E_GSE292324_heatmap_top25_symbols_n.png', width=1600, 
height=1400, res=220)
    pheatmap::pheatmap(z292324_25_sym, annotation_col = 
ann_292324_25, show_rownames = TRUE, fontsize_row=7, fontsize_col=8,
           main='GSE292324: Top 25 DEGs (Symbols, row Z) [n=9/
group]')
    dev.off()
  }
  if (exists("z292050_25_sym") && exists("ann_292050_25")){
    png('Fig1F_GSE292050_heatmap_top25_symbols_n.png', width=1600, 
height=1400, res=220)
    pheatmap::pheatmap(z292050_25_sym, annotation_col = 
ann_292050_25, show_rownames = TRUE, fontsize_row=7, fontsize_col=8,
           main='GSE292050: Top 25 DEGs (Symbols, row Z) [n=9/
group]')
    dev.off()
  }
}
```
