# Supplementary material for: Targeted therapies reshape extracellular matrix remodeling and microenvironmental regulation in pediatric acute myeloid leukemia
Source: Discov Oncol. 2026 Feb 21;17:491. doi: 10.1007/s12672-026-04617-w (PMC13031502; doi:10.1007/s12672-026-04617-w)
Supplement: Supplementary file 3 — Additional file 3. [file 12672_2026_4617_MOESM3_ESM.docx]

**Supplementary Materials**

**Manuscript Title**: Targeted Therapies and Microenvironmental Modulation in Pediatric AML: An Integrative Transcriptomic Analysis


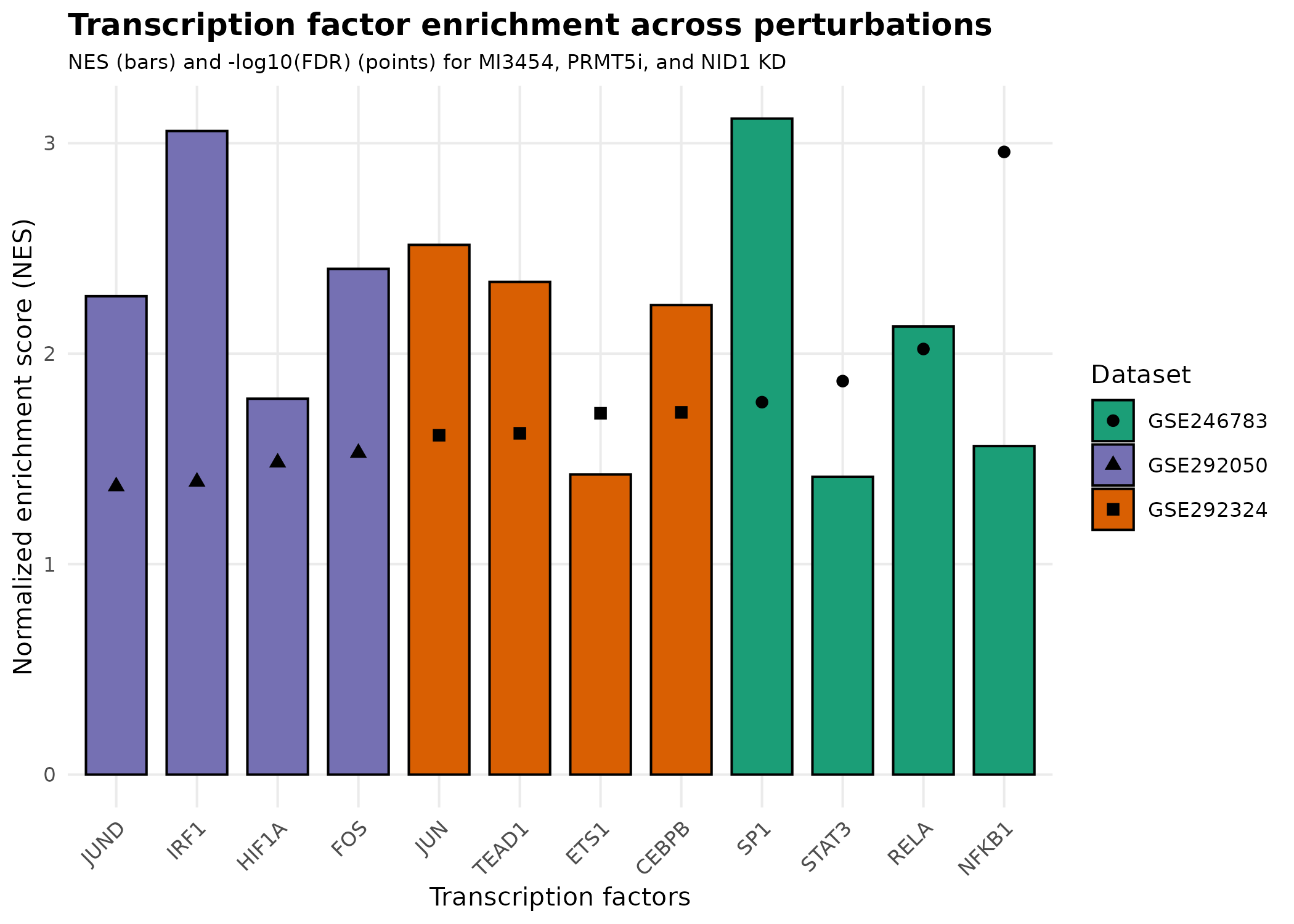


**Supplementary Figure S5.** ***Transcription factor enrichment across perturbation datasets.***

Bar plot showing normalized enrichment scores (NES) for the top transcription factors identified by TF–target enrichment analysis (Enrichr; ChEA/ENCODE) in GSE246783 (MI3454 therapy), GSE292324 (PRMT5 inhibition), and GSE292050 (NID1 knockdown). Overlaid points represent –log10(FDR) values for each transcription factor. Together with Table 4, this analysis highlights NF-κB (NFKB1/RELA), STAT3, SP1, and CEBPB as core regulators, along with ECM- and stress-responsive factors including ETS1, TEAD1, FOS, HIF1A, IRF1, and JUND.


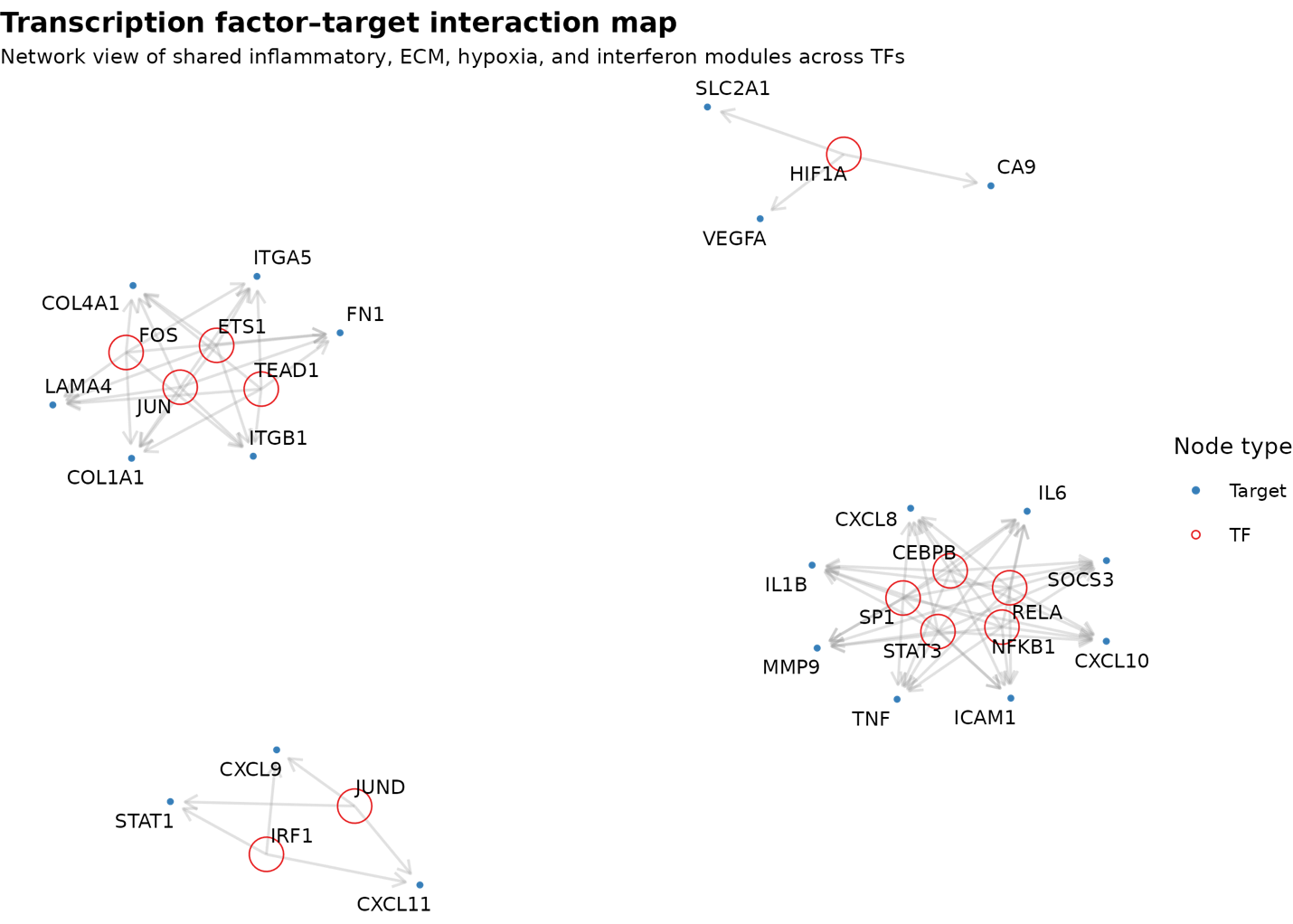


**Supplementary Figure S6. Transcription factor–target interaction network.**

Network visualization of predicted and experimentally supported transcription factor (TF)–target gene relationships derived from the integrative analysis of pediatric AML perturbation datasets. TFs are represented as larger, distinctively colored nodes, and target genes as smaller nodes. Directed edges denote regulatory interactions from each TF to its putative targets, weighted by interaction confidence and shared enrichment across MI3454 treatment, PRMT5 inhibition, and NID1 knockdown models. Highly connected hubs reflect key regulatory modules governing **ECM/MMP remodeling**, **immune and inflammatory signaling**, and **chromatin/splicing regulation**. This network summarizes the central regulatory architecture underlying convergent transcriptomic responses in pediatric AML
