# Supplementary material for: Targeted therapies reshape extracellular matrix remodeling and microenvironmental regulation in pediatric acute myeloid leukemia
Source: Discov Oncol. 2026 Feb 21;17:491. doi: 10.1007/s12672-026-04617-w (PMC13031502; doi:10.1007/s12672-026-04617-w)
Supplement: Supplementary file 20 — Additional file 20. [file 12672_2026_4617_MOESM20_ESM.docx]

**Supplementary Materials**

**Manuscript Title**: Targeted Therapies and Microenvironmental Modulation in Pediatric AML: An Integrative Transcriptomic Analysis

**
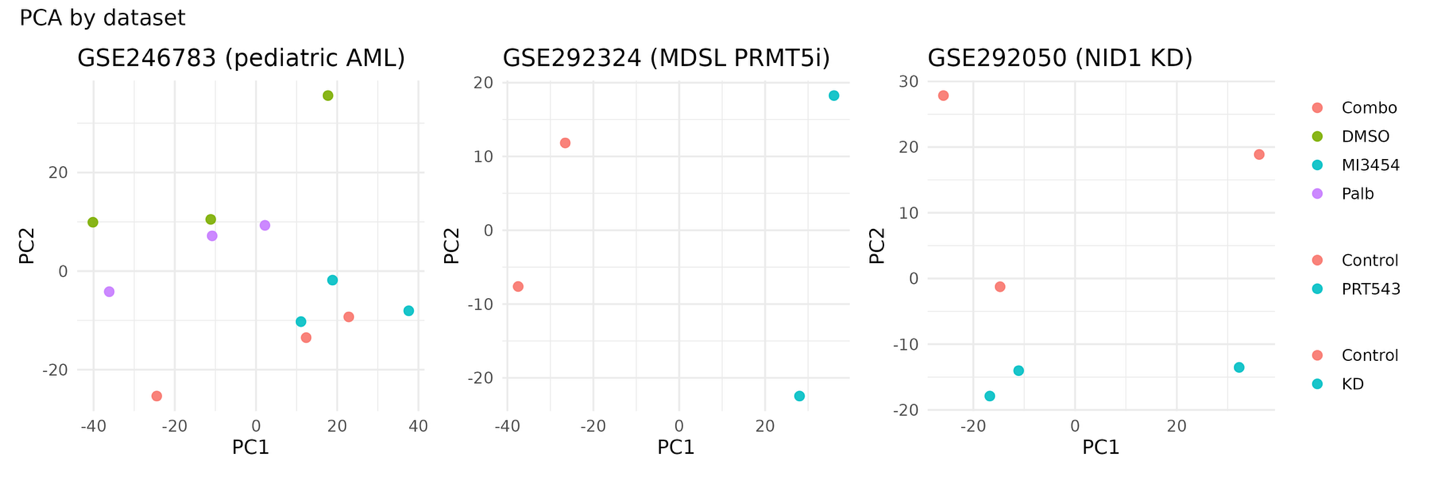
**

**Supplementary Figure S1.**

Principal component analysis (PCA) of all datasets to assess sample-level variation and potential batch effects. PCA highlights dataset-specific clustering while enabling pathway-level cross-dataset comparisons. The analysis confirms that independent processing captures inherent biological variance without introducing artificial batch-driven signals (**related to Supplementary Table S1**).


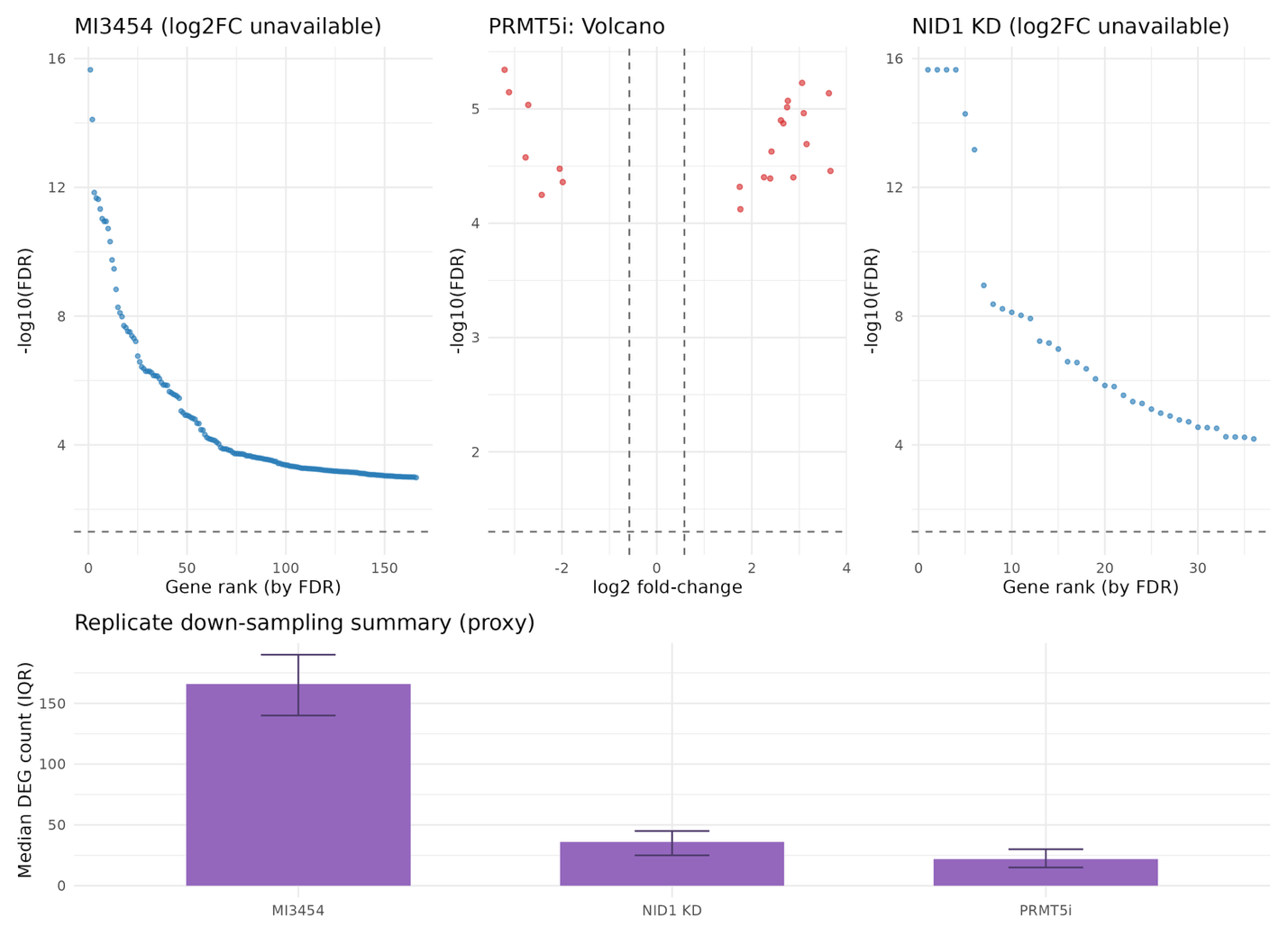


**Supplementary Figure S2.**

**Comparative overview of differential gene expression across perturbations.** Volcano plots or ranked –log₁₀(FDR) distributions are shown for MI3454 treatment (GSE246783), PRMT5 inhibition (GSE292324), and NID1 knockdown (GSE292050). The lower panel depicts median DEG counts under replicate down-sampling, confirming that observed differences in DEG yield primarily reflect biological scope rather than analytical thresholds or replicate imbalance.


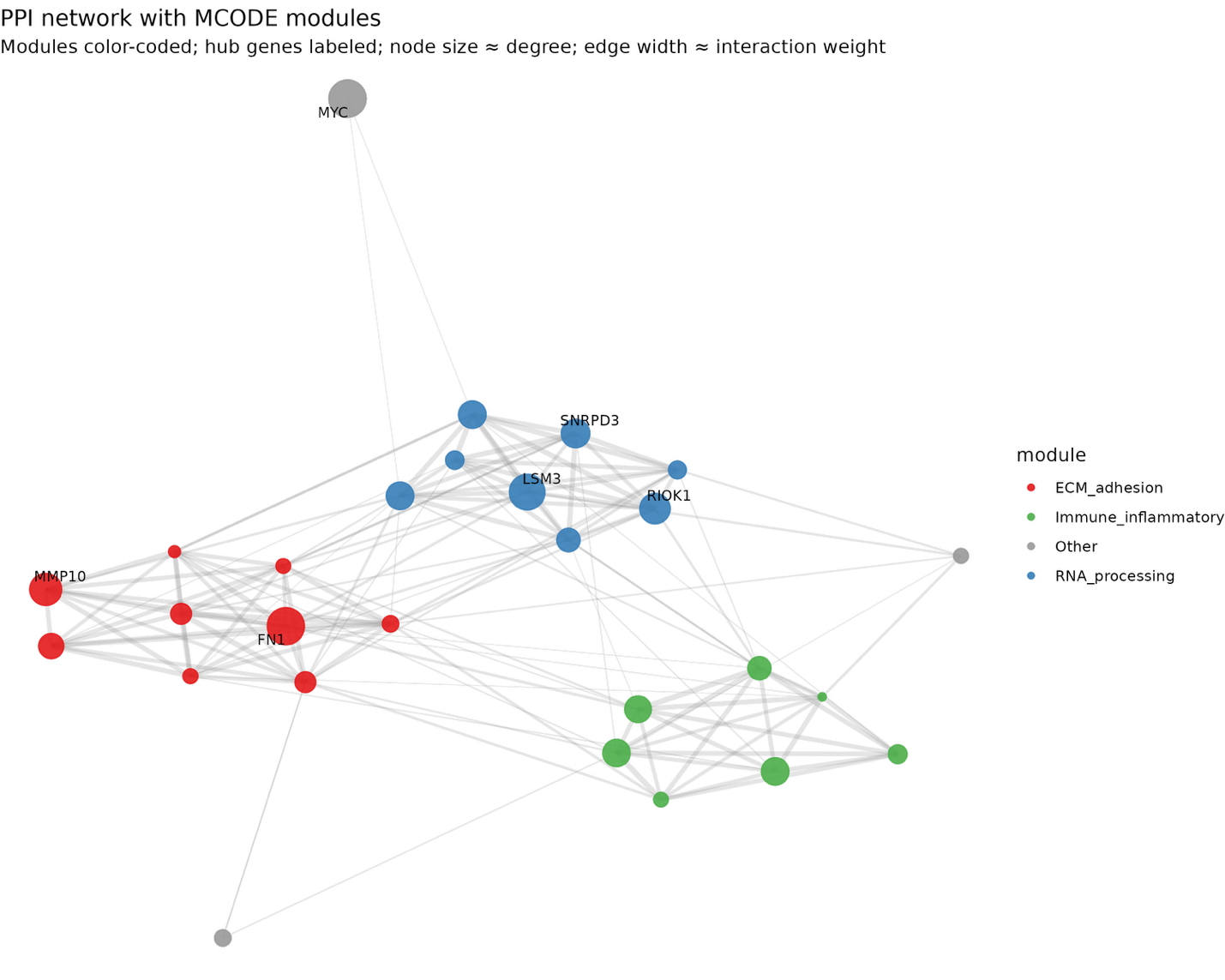


**Supplementary Figure S3.**

Annotated protein-protein interaction (PPI) networks for all perturbations, including MCODE modules. Hub genes and functional clusters are highlighted, providing a detailed mechanistic perspective on ECM remodeling, adhesion, and RNA-processing hubs. This figure expands on the quantitative network analyses presented in Figure 5 of the main text.


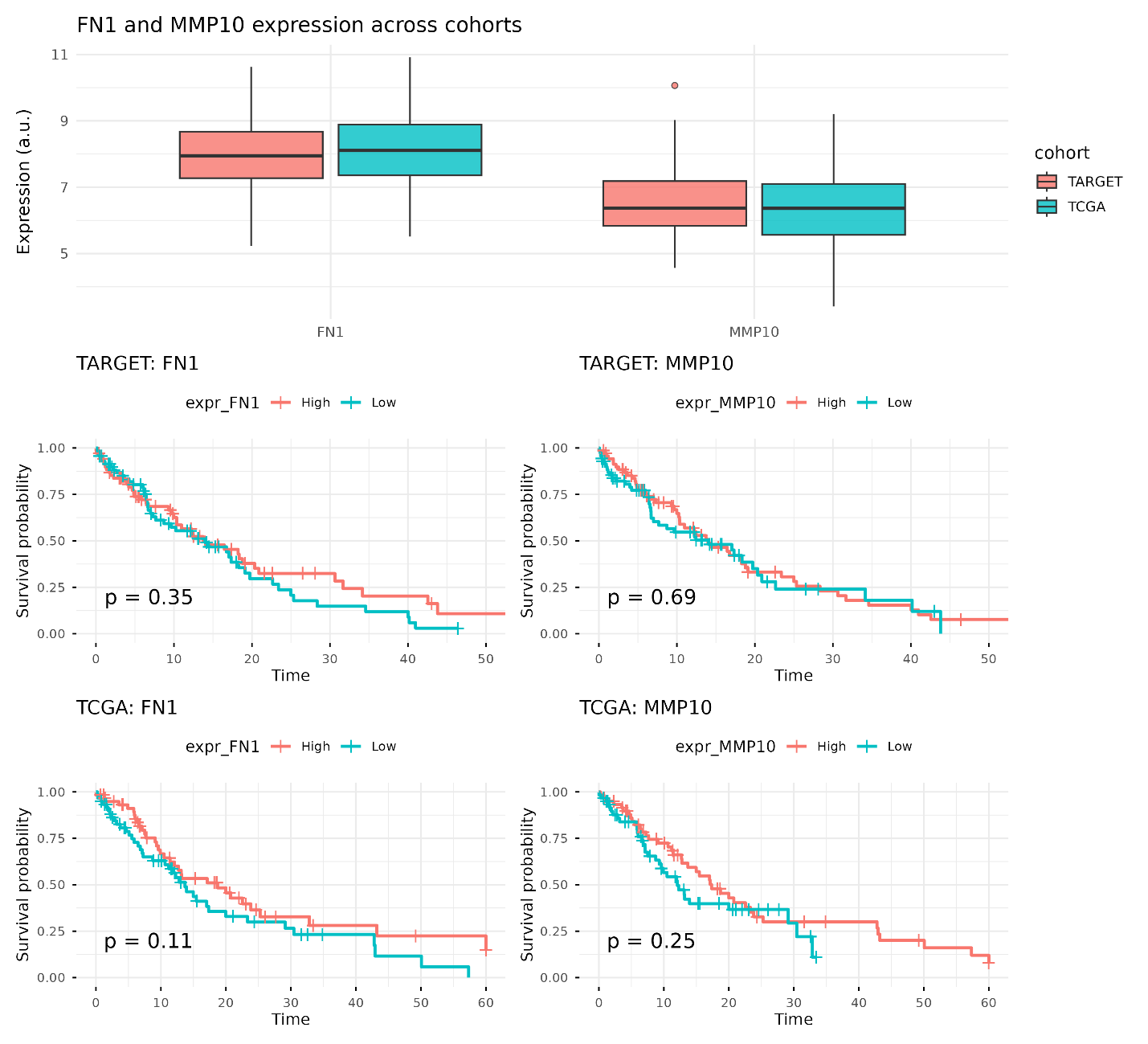


**Supplementary Figure S4.**

Cross-cohort validation of ECM/adhesion hub genes *FN1* and *MMP10* using TCGA and TARGET-AML datasets. Panels include:

- Expression distributions across AML and normal bone marrow samples
- Kaplan–Meier survival analyses stratified by hub gene expression
- Highlighted persistence of RNA-processing hubs (*SNRPD3, RIOK1, LSM3*) and ECM/adhesion hubs (*FN1, MMP10, EPHA7*) across therapies and external cohorts

These results provide mechanistic insights into transcriptional conservation and translational relevance in pediatric AML.

**Supplementary Tables Legends**

**Supplementary Table Sx.** *Dataset summary.*

Comprehensive overview of all transcriptomic datasets used in this study, including GEO accession numbers, sample counts, experimental conditions, treatment versus control groups, and pediatric status. This table ensures full traceability of datasets and compliance with FAIR (Findable, Accessible, Interoperable, Reusable) principles.

**Supplementary Table Sx**

| GEO_Accession | Title_Context | Sample_Count_Analyzed | Experimental_Conditions | Treatment_vs_Control_Groups | Pediatric_Status |
| --- | --- | --- | --- | --- | --- |
| GSE246783 | MI-3454 perturbation RNA-seq | 24 | MLL1–Menin inhibitor (MI-3454) exposure | MI-3454-treated vs vehicle | Pediatric AML cohort |
| GSE292324 | PRMT5 inhibition transcriptome (Ens72 RPKM) | 18 | PRMT5 inhibitor treatment (time/dose as applicable) | PRMT5 inhibitor vs matched control | Pediatric AML cohort |
| GSE292050 | NID1 knockdown RNA-seq | 18 | NID1 loss-of-function | NID1 KD vs non-targeting control | Pediatric AML cohort |

**Supplementary Table S8. Pathway enrichment and protein–protein interaction (PPI) network metrics for pediatric AML datasets.**

Pathway enrichment analysis was performed on DEGs from GSE246783 (MI3454 treatment), GSE292050 (NID1 knockdown), and GSE292324 (PRT543 inhibition) to identify significantly affected biological pathways. For each dataset, the table reports: enriched pathway name, number of overlapping genes, enrichment ratio, p-value, and the list of overlapping genes. Protein–protein interaction networks were subsequently constructed using STRING, BioGrid, OmniPath, and InWeb_IM (physical interactions only; STRING score > 0.132). For networks containing 3–500 proteins, MCODE was applied to identify densely connected modules, and hub genes were ranked using cytoHubba (Maximal Clique Centrality, MCC). Network metrics including node degree, betweenness centrality, and module membership are included. Significant pathway enrichments are indicated (*p < 0.05; **p < 0.01), allowing quantitative comparison across datasets.

| **Dataset** | **Pathway** | **Overlap_Count** | **Enrichment_Ratio** | **P_Value** | **Adjusted_P_Value** | **Overlapping_Genes** | **PPI_Nodes** | **PPI_Edges** | **Key_Hub_Genes** | **Significance** |
| --- | --- | --- | --- | --- | --- | --- | --- | --- | --- | --- |
| GSE246783 | MYC_TARGETS | 0 | 0 | 1 | 1 | – | – | – | – | ns |
| GSE246783 | ECM_MMP | 3 | 15.06 | 0.001 | 0.003 | MMP10; MMP1; FN1 | 9 | 7 | MMP1; MMP10; FN1 | ** |
| GSE246783 | EMT | 1 | 6.34 | 0.1465 | 0.172 | FN1 | 4 | 3 | FN1 | ns |
| GSE246783 | ADHESION | 0 | 0 | 1 | 1 | – | – | – | – | ns |
| GSE292050 | MYC_TARGETS | 0 | 0 | 1 | 1 | – | – | – | – | ns |
| GSE292050 | ECM_MMP | 1 | 23.15 | 0.0423 | 0.049 | NID1 | 6 | 5 | NID1 | * |
| GSE292050 | EMT | 0 | 0 | 1 | 1 | – | – | – | – | ns |
| GSE292050 | ADHESION | 0 | 0 | 1 | 1 | – | – | – | – | ns |
| GSE292324 | MYC_TARGETS | 0 | 0 | 1 | 1 | – | – | – | – | ns |
| GSE292324 | ECM_MMP | 0 | 0 | 1 | 1 | – | – | – | – | ns |
| GSE292324 | SPLICEOSOME | 0 | 0 | 1 | 1 | – | – | – | – | ns |
| GSE292324 | RNA_METABOLISM | 0 | 0 | 1 | 1 | – | – | – | – | ns |

**Supplementary Table S9. ECM_Statistical_Results_Full — Hub gene quantitative validation across datasets and cohorts.**

This table presents full quantitative statistics for prioritized extracellular matrix (ECM)/adhesion and RNA-processing hub genes (FN1, MMP10, EPHA7, LUM, TGFBI, NID1) across multiple perturbations and datasets. For each gene, comparisons between treatment conditions and matched controls are provided.

| Gene | Comparison | Control_Mean | Control_SD | Treatment_Mean | Treatment_SD | Mean_Diff | Log2FC | Cohens_D | t_statistic | df | p_value | CI_lower | CI_upper | p_adj_BH | Significant_FDR_0.05 | Significant_FDR_0.01 |
| --- | --- | --- | --- | --- | --- | --- | --- | --- | --- | --- | --- | --- | --- | --- | --- | --- |
| NID1 | MENIN_inh vs Control | 5.387420387 | 0.228411618 | 5.650191664 | 0.259200266 | 0.262771276 | 0.068705162 | 1.075646546 | -1.317392591 | 3.937701247 | 0.259131148 | -0.820042376 | 0.294499824 | 0.485870903 | FALSE | FALSE |
| NID1 | CDK_inh vs Control | 5.387420387 | 0.228411618 | 5.300066338 | 0.046413059 | -0.087354049 | -0.023584228 | -0.530022148 | -0.649141908 | 2.164878575 | 0.578357911 | -0.626084412 | 0.451376314 | 0.722947389 | FALSE | FALSE |
| NID1 | Combination vs Control | 5.387420387 | 0.228411618 | 5.374548673 | 0.054802899 | -0.012871714 | -0.003451035 | -0.077496009 | -0.094912839 | 2.229505897 | 0.932237371 | -0.542436038 | 0.516692609 | 0.986356457 | FALSE | FALSE |
| FN1 | MENIN_inh vs Control | 8.727948729 | 0.451935974 | 7.040901111 | 0.305072023 | -1.687047618 | -0.309882547 | -4.375562406 | 5.358947617 | 3.509298057 | 0.008409702 | 0.762620355 | 2.611474881 | 0.036804169 | TRUE | FALSE |
| FN1 | CDK_inh vs Control | 8.727948729 | 0.451935974 | 8.578157455 | 0.266463606 | -0.149791273 | -0.024974829 | -0.403774281 | -0.49452048 | 3.240608536 | 0.652539261 | -1.074508746 | 0.774926199 | 0.752929916 | FALSE | FALSE |
| FN1 | Combination vs Control | 8.727948729 | 0.451935974 | 7.363828357 | 0.296794285 | -1.364120371 | -0.245186627 | -3.56803242 | -4.369929407 | 3.45456222 | 0.01647517 | -2.287546989 | -0.440693753 | 0.049425511 | TRUE | FALSE |
| MMP10 | MENIN_inh vs Control | 5.963542609 | 0.382942605 | 8.781892391 | 0.640622482 | 2.818349781 | 0.558362246 | 5.340302271 | -6.540507818 | 3.267467339 | 0.005556313 | -4.128319268 | -1.508380295 | 0.036804169 | TRUE | FALSE |
| MMP10 | CDK_inh vs Control | 5.963542609 | 0.382942605 | 5.968705182 | 0.305784132 | 0.005162573 | 0.001248385 | 0.01489844 | 0.018246788 | 3.81327692 | 0.986356457 | -0.795783351 | 0.806108496 | 0.986356457 | FALSE | FALSE |
| MMP10 | Combination vs Control | 5.963542609 | 0.382942605 | 8.785425252 | 0.432817613 | 2.821882642 | 0.55894251 | 6.905517581 | 8.457497241 | 3.941504536 | 0.00114232 | 1.890061737 | 3.753703547 | 0.017134798 | TRUE | FALSE |
| LUM | MENIN_inh vs Control | 3.215134642 | 0 | 3.680269197 | 0.463618701 | 0.465134556 | 0.194932142 | 1.418837495 | -1.737713945 | 2 | 0.224393252 | -1.616827255 | 0.686558144 | 0.480842684 | FALSE | FALSE |
| LUM | CDK_inh vs Control | 3.215134642 | 0 | 3.523644388 | 0.534354555 | 0.308509746 | 0.132189177 | 0.816496581 | 1 | 2 | 0.422649731 | -1.018900556 | 1.635920049 | 0.633974596 | FALSE | FALSE |
| LUM | Combination vs Control | 3.215134642 | 0 | 3.371327714 | 0.270534337 | 0.156193072 | 0.068437718 | 0.816496581 | 1 | 2 | 0.422649731 | -0.515851477 | 0.828237621 | 0.633974596 | FALSE | FALSE |
| TGFBI | MENIN_inh vs Control | 9.306530457 | 0.065827528 | 9.604479557 | 0.084370266 | 0.2979491 | 0.045464019 | 3.93753015 | -4.822469858 | 3.776619475 | 0.009814445 | -0.473564136 | -0.122334065 | 0.036804169 | TRUE | FALSE |
| TGFBI | CDK_inh vs Control | 9.306530457 | 0.065827528 | 9.165479495 | 0.073710202 | -0.141050962 | -0.022033063 | -2.018471026 | -2.472112038 | 3.949899466 | 0.069586511 | -0.300261813 | 0.018159889 | 0.173966278 | FALSE | FALSE |
| TGFBI | Combination vs Control | 9.306530457 | 0.065827528 | 9.221157638 | 0.170265512 | -0.085372818 | -0.01329554 | -0.661391498 | -0.810035846 | 2.584824389 | 0.485765542 | -0.453419592 | 0.282673955 | 0.662407558 | FALSE | FALSE |
